# Selective Negative Allosteric Modulation Of Metabotropic Glutamate Receptors – A Structural Perspective of Ligands and Mutants

**DOI:** 10.1038/srep13869

**Published:** 2015-09-11

**Authors:** Kasper Harpsøe, Vignir Isberg, Benjamin G. Tehan, Dahlia Weiss, Angela Arsova, Fiona H. Marshall, Hans Bräuner-Osborne, David E. Gloriam

**Affiliations:** 1 Department of Drug Design and Pharmacology, Faculty of Health and Medical Sciences, University of Copenhagen, Jagtvej 162, 2100 Copenhagen, Denmark; 2 Heptares Therapeutics Ltd, BioPark, Broadwater Road, Welwyn Garden City, AL7 3AX, UK

## Abstract

The metabotropic glutamate receptors have a wide range of modulatory functions in the central nervous system. They are among the most highly pursued drug targets, with relevance for several neurological diseases, and a number of allosteric modulators have entered clinical trials. However, so far this has not led to a marketed drug, largely because of the difficulties in achieving subtype-selective compounds with desired properties. Very recently the first crystal structures were published for the transmembrane domain of two metabotropic glutamate receptors in complex with negative allosteric modulators. In this analysis, we make the first comprehensive structural comparison of all metabotropic glutamate receptors, placing selective negative allosteric modulators and critical mutants into the detailed context of the receptor binding sites. A better understanding of how the different mGlu allosteric modulator binding modes relates to selective pharmacological actions will be very valuable for rational design of safer drugs.

Glutamate is the major excitatory neurotransmitter and has fast synaptic action via ionotropic glutamate receptors and modulatory actions through metabotropic glutamate (mGlu) receptors in the brain (mGlu_1–5_ and mGlu_7–8_) and retina (mGlu_6_ and mGlu_8_)^1^. The mGlu receptors belong to the class C G protein-coupled receptors (GPCRs) and comprise eight receptors divided into three groups; I (mGlu_1_ and mGlu_5_), II (mGlu_2_ and mGlu_3_) and III (mGlu_4_ and mGlu_6–8_). Like the other class C GPCRs, the mGlu receptors, function as homo- or heterodimers^2^, and are composed of three distinct functional topological domains. Glutamate binds within a large extracellular Venus flytrap domain and a cysteine-rich domain communicates the signal to a seven transmembrane (7TM) domain^3^,^4^,^5^. Even though several crystal structures of the mGlu receptor Venus flytrap domain has been available for more than a decade^3^, only few subtype-selective orthosteric ligands have been reported^6^,^7^ due to the very high conservation of the orthosteric binding site^8^. Allosteric modulation in the 7TM domain^9^ has proved a lot more tractable and hundreds of subtype-selective modulators have been reported, most abundantly for mGlu_2_^10^, mGlu_4_^11^,^12^ and mGlu_5_^13^,^14^.

Clinical and pre-clinical studies have associated the mGlu receptors with a broad range of neurological diseases^1^,^4^,^5^,^15^,^16^ (Table 1), and notably, mGlu_5_ is among the top four most pursued drug targets by pharmaceutical companies^17^. mGlu allosteric modulators have been reported in patent applications^10^,^12^,^14^, and clinical trials for e.g. mGlu_2_^10^ and mGlu_5_^14^. The distribution of selective negative allosteric modulators (NAMs) reflects the potential therapeutic application of their targets being most frequent for group I receptors^14^,^18^, intermediate for group II receptors^16^,^19^ and few for group III limited to the mGlu_7_^20^,^21^ subtype. Future mGlu receptor drug design holds substantial opportunities, but also challenges as cross activity is often reported for allosteric ligands, for example mGlu_4_ positive allosteric modulators (PAMs) act as mGlu_1_ or mGlu_5_ NAMs^5^,^11^,^22^. Thus, the activity needs to be fine-tuned and balanced towards the right set of receptor groups or subtypes^23^.

Academic groups^24^,^25^,^26^, Pfizer^27^, Roche^28^,^29^, Novartis^30^ and Merck^31^ have employed receptor structure-based design of selective mGlu receptor NAMs. The ligand-receptor binding modes were refined using mutagenesis and structure-activity data, but the underlying receptor models were built on class A GPCR templates with less than 15% sequence identity. A recent breakthrough in crystallography have now presented the first experimental structures of the mGlu_1_^9^ and mGlu_5_^32^ 7TM domains in complex with the NAMs, FITM and mavoglurant, respectively. In this light, we have to re-evaluate our understanding of reported mGlu receptor mutational effects and ligand structure-activity relationships. The combined structural, pharmacological and chemistry data now present an unprecedented platform for structure-based design of new mGlu receptor NAMs.

Herein, we combine the accumulated mutagenesis data (Supplementary Table 1), selective NAMs and receptor 7TM domain structures (crystallized and new high-resolution models) to explain the modulator-target interactions that determine pharmacology. This is the first comprehensive comparison of the allosteric 7TM domain binding pocket for all clinically targeted mGlu receptor subtypes. It presents an outline of the not yet exploited unique residue hotspots within the binding sites that offer the most viable contact points for achieving subtype-selective NAMs.

## Results

### NAM-mGlu binding modes can explain selectivity and mutant effects

**Figure 1:**
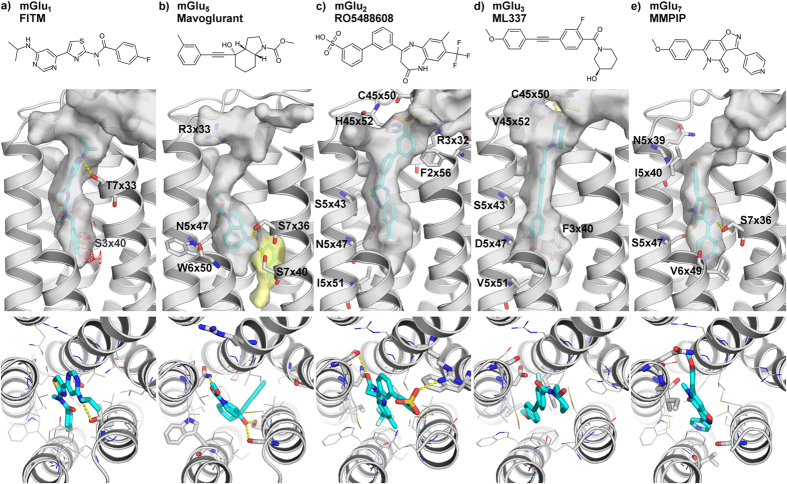
Receptor binding of mGlu receptor subtype-selective NAMs. NAM 2D structures and binding mode side- and top-views in the crystal structure complexes of the group I (**a**) mGlu_1_-FITM and (**b**) mGlu_5_-mavoglurant complexes; and new high-precision models of the group II (**c**) mGlu_2_-RO5488608, (**d**) mGlu_3_-ML337 and group III (**e**) mGlu_7_-MMPIP binding. Except for a deep mGlu5 sub-pocket (yellow surface in (b)), the mGlu receptor binding cavities are similar in shape and size. The Figure highlights the binding site surfaces, hydrogen bonds (yellow dashed lines) and a hydrophilic area (red mesh) near mGlu1 Ser668^3x40^. Throughout this manuscript, the generic residue positions in the TM helices are denoted with the structure-based GPCRdb numbering system that takes helix bulges and constrictions into account^60^. mGlu_4_, mGlu_6_ and mGlu_8_, are not included in this figure due to lack of subtype-selective NAMs. The 2D chemical structures were prepared with MarvinSketch 6.2.1, 2014, ChemAxon and the 3D images with the PyMOL Molecular Graphics System, version 1.7 Schrödinger, LLC.

**Figure 2:**
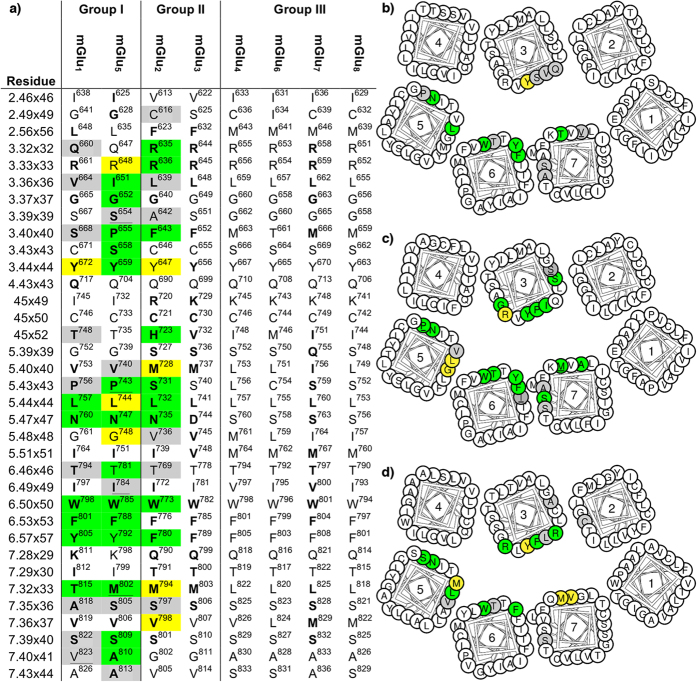
Residues in the mGlu receptor allosteric 7TM domain binding pockets. (**a**) Sequence alignment of the mGlu receptor binding pocket residues. Helix box diagrams of (**b**) mGlu_1_, (**c**) mGlu_5_ and (**d**) mGlu_2_ displaying mutated residue positions from the extracellular side^61^. The generic residue numbers combine the sequence-based class C scheme by Pin *et al.*^62^ and the structure-based GPCRdb scheme^60^. Bold amino acids (**a**) and positions (**b-d**) have Cα or side chain atoms within 5 Å of the bound NAMs in Figure 1. Colored residues have been mutated and tested for effect on NAM binding and/or function, yellow: ≥3-fold and <10-fold, green: ≥10-fold effect on binding and/or potency for any tested NAM, grey: < 3-fold effect on any of the tested modulators; and underlined grey/green indicates that no/marked effect is based on qualitative data (Supplementary Table 1). Of note, the accumulated literature mutagenesis data agrees very well with the new crystallographic data in the pinpointing of ligand-binding residues.

To explain subtype-selectivity, we have conducted the first comprehensive comparison of mGlu NAM binding sites and modes. Figure 1 shows the mGlu_1_ and mGlu_5_ crystal structure complexes complemented by high-precision models for mGlu_2_ (RO5488608^29^), mGlu_3_ (ML337^19^) and mGlu_7_ (MMPIP^33^) generated by homology modeling and induced-fit docking. This modeling approach was validated on the two crystal structure complexes, and could successfully (re-)produce very similar poses (Supplementary Figure 1). Even though the NAMs are structurally different and subtype-selective^9^,^32^, their binding sites are remarkably similar in shape and size. The group II and III mGlu receptors have a larger overall fraction of flexible hydrophobic residues that could adapt to different modulator scaffolds. Figure 2a outlines the corresponding residues in the binding sites, which display rather high sequence similarities, 68–74%, but unique residues exist that can be correlated to the selectivity of known NAMs (below). Furthermore as also described below for each mGlu group, mapping of the accumulated *in vitro* mutant data (Fig. 2a–d, Supplementary Table 1) could provide structural explanations of the observed effects, while also in many cases support the docking experiments.

#### Group I selective NAM binding mode

FITM occupies its site fully in mGlu_1_, while mavoglurant only fills the bottom part in mGlu_5_ (Fig. 1a,b), including a unique deep small sub-pocket^9^,^32^ (Supplementary Fig. 2). The FITM-mGlu_1_ interactions are all hydrophobic, except for a hydrogen bond between the amine derivate of the pyrimidine 5-position with the side chain of Thr815^7×33^. However, our induced fit docking of FITM into a mGlu_1_ homology model shows that a flip of the *N*-Methylamide moiety allows for a similar binding mode that picks up a hydrogen bond between the FITM carbonyl and Asn760^5×47^. This pose fits the crystal structure electron density as well as the one presented in the PDB structure (Supplementary Figure 1b), signifying that the docking pose is as likely a binding mode or even more likely due to the additional hydrogen bond. The mavoglurant-mGlu_5_ interactions include hydrogen bonds from the carbamate to Asn747^5×47^ and from the hydroxyl to Ser805^7×36^ and Ser809^7×40^, whereas the methylphenyl ring occupies the unique sub-pocket, which is situated between TM2, 3 and 7. The binding site residues include a large number of reported mutants with effect on modulators for mGlu_1_^9^,^34^,^35^ and mGlu_5_^24^,^26^,^36^ (Fig. 2a,b, Supplementary Table 1).

#### Group II selective NAM binding mode

The subtype-preferring/selective NAMs, RO5488608^29^ and ML337^19^, display a tight fit within the mGlu_2_ and mGlu_3_ binding pockets, respectively (Fig. 1c,d). Among the top ten docking poses of RO5488608, we identified five that were unique, three of these were in contact with all four residues shown by mutations to affect the RO5488608 potency^29^ and the other two were disregarded as likely binding poses. The remaining poses displayed similar scoring values and low conformational energy penalties, and we selected the 4^th^ ranking pose as this has the most optimal polar interactions to the receptor (Supplementary Table 2). Based on this pose, the RO5488608-mGlu_2_ polar interactions are proposed to include amide carbonyl-Ser731^5×43^ and nitrogen-Asn735^5×47^ hydrogen bonds, as well as sulphonic acid salt bridges/hydrogen bonds to the side chains of His723^45×52^, Arg635^3×32^ and the Cys721^45.50^ backbone. Furthermore, aromatic contacts are proposed to Phe623^2×56^, Phe643^3×40^, Phe776^6×53^ and Phe780^6×57^.

ML337 docking also resulted in five unique poses among top the ten, all with low conformational energy penalties, but only the selected pose was consistent with the sparse structure-activity relationship (SAR) information^19^ and at the same time had better hydrogen bonding interactions and markedly better Emodel score (Supplementary Table 3). This ML337-mGlu_3_ binding model shows a hydroxyl-Cys730^45×50^ backbone hydrogen bond, and contacts with the same four aromatic residues as RO5488608 in mGlu_2_. Moreover, both RO5488608 and ML337 are close to the second extracellular loop (ECL2), which contains a cysteine bridge to TM3 conserved in all GPCR classes A–C. In the two former classes, this loop has been shown to be involved in ligand binding, activation and regulation^37^; and our results could suggest that it has an effect on allosteric modulation in class C GPCRs.

#### Group III selective NAM binding mode

Docking MMPIP to our mGlu_7_ homology model resulted only in two different output poses and since our objective pose selection criteria and the sparse SAR^21^ did not allow for a clear cut selection we selected the pose that offered the most likely explanation for the experimentally observed selectivity; a hydrogen bond to Ser763^5×47^ from the carbonyl in MMPIP (Supplementary Table 4). Additionally, the MMPIP-mGlu_7_ polar interactions include a hydrogen bond from the isoxazole nitrogen to Ser828^7×36^ (Fig. 1e). MMPIP is not in contact with ECL2, but the methoxy substituent is near Gln755^5×39^ in an area where the mGlu_5_ structure contains a water molecule^32^ indicating the potential of an additional polar interaction. At the bottom of the binding pocket, a cluster of hydrophobic residues accommodates the pyridine ring. Only sparse SAR data is available for MMPIP, but the binding mode is in agreement with the information from these close analogues^21^ (Supplementary Table 4).

### mGlu group and subtype-unique residues – the hotspots available to mediate selective ligand interactions

**Figure 3:**
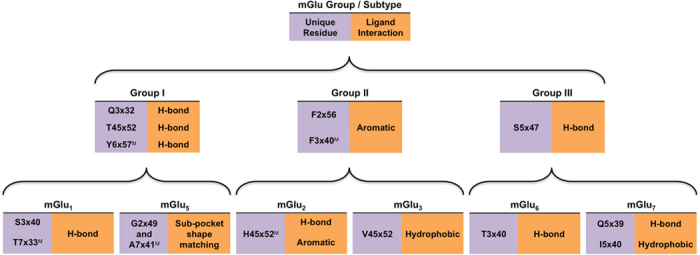
mGlu group and subtype unique residues suggested as hotspots for selectivity and their possible ligand interactions. The comparative binding pocket analyses (Figs. 1-2) pinpointed six group-specific and nine subtype-specific residues that can be exploited to gain selectivity. Each residue is listed together with the potential ligand interactions that can result in selectivity. ^M^ denotes that this residue has already been validated by mutagenesis experiments as having effect on ligand binding/potency. No unique residues were found for the group III mGlu_4_ and mGlu_8_ receptors.

As the mGlu allosteric 7TM binding pockets are very similar in shape and size (above), selectivity primarily has to be achieved through interactions with unique residue side chains. We have compared all mGlu receptors, to identify such selectivity hotspots on both the group- and subtype levels (Fig. 3). Some of these positions have already been mutated with an effect on ligand binding/potency (Supplementary Table 1), whereas more than half represents new potential interactions. Figure 3 provides a graphical summary of the identified selectivity hotspots within the mGlu groups and subtypes, together with the type of interaction expected to result in selectivity and the point mutations that could validate the effect on modulator potency and selectivity.

#### Group I selectivity hotspots

Group I mGlu receptors are distinguished from the two other groups by three hydrophilic residues in the top of the binding site, Gln3×32, Thr45×52 and Tyr6×57. These residues do not interact with mavoglurant (Fig. 1a) or FITM (Fig. 1b), but could form hydrogen bonds with other NAMs and PAMs specifically targeting this group. In mGlu_1_, Thr815^7×33^ and Ser668^3×40^ stand out as potential selectivity hotspots. Mutation of Thr815^7×33^ has previously been shown to influence modulator activity^34^,^38^. This is explained by a FITM hydrogen bond^9^ (Fig. 1a), which cannot be formed in other mGlu receptors as they have hydrophobic residues (Fig. 2). Ser668^3×40^ could also form a hydrogen bond and, except for a threonine in mGlu_6_, all other subtypes contain hydrophobic residues in this position. Our alternative binding mode of FITM from induced fit docking to a mGlu_1_ model (Supplementary Figure 1a) places the FITM carbonyl within 3.3 Å of the Ser668^3×40^ hydroxyl hinting a weak electrostatic interaction, which may correspond to the observed weak effect of S668^3×40^P mutation on FITM function^9^. However, a more favourable hydrogen bond to Ser668^3×40^ may be achieved by 2- or 3-substitution of the FITM fluorobenzamide moiety with a hydrogen bond donor or acceptor as exemplified by docking of a 2-pyridine analogue of FITM (Supplementary Figure 3). Taken together, Ser668^3×40^ and Thr815^7×33^ represent hydrogen bonding partners with the ability to induce mGlu_1_ selectivity.

In mGlu_5_, the most characteristic feature is the unique deep sub-pocket with a tight fit to the mavoglurant acetylene-aromate-moiety (Fig. 1b and Supplementary Fig. 2). Many additional selective mGlu_5_ modulators, such as MPEP^13^, share this moiety but there are also modulators, like fenobam, with other scaffolds that occupy this pocket^28^. This pocket has a very narrow entrance in all other subtypes, but is accessible in mGlu_5_ because of the smaller side chains of Gly628^2×49^, Pro655^3×40^ and Ala810^7×41^. In our mGlu_1_ and group II mGlu receptors models this sub-pocket is blocked by Val823^7×41^ and Phe3×40, respectively, which is confirmed by the fact that mGlu_5_ A810^7×41^V and P655^3×40^F mutants^24^,^26^,^28^,^30^ abolish NAM inhibition. Also Group III mGlu receptors contain residues with longer side chains; in positions 2×49, 3×40, 7×37 and 7×44 (Fig. 2 and Supplementary Figure 2); expected to occlude the entrance and parts of the deep mGlu_5_ sub-pocket. An exception could be MPEP, an mGlu_5_ NAM with weak activity on mGlu_4_^12^,^22^. MPEP is small and it is possible that the flexible Met663^3×40^ in mGlu_4_ may allow access to the sub-pocket, but it could also have another binding mode/site. Apart from the binding within the deep sub-pocket, a mavoglurant-Ser805^7×36^ hydrogen bond contributes further to the mGlu_5_ selectivity over mGlu_1_^32^. Taken together, our comparative sequence analysis and the supporting experimental data pinpoint the deep sub-pocket as the prime site to achieve mGlu_5_ subtype selectivity, which can be further improved by hydrogen bonding to Ser805^7×36^.

#### Group II selectivity hotspots

The group II mGlu receptors have unique phenylalanine residues in positions 2×56 and 3×40 (Fig. 2) that may be exploited to induce selectivity. In our mGlu_2_ docking, Phe623^2×56^ formed an edge-to-face π-π interaction with RO5488608, whereas Phe643^3×40^ displayed only weak van der Waals interactions to the trifluoromethyl moiety, in line with reported F643A^3×40^ mutagenesis results^29^ (Fig. 1c). However, the potential of Phe643^3×40^ in mediating selectivity is supported by its reported potentiation of mGlu_2_ selective PAMs^39^ and our suggested binding mode of ML337 (Fig. 1d). In mGlu_3_ Phe652^3×40^ is predicted to form the strongest contact, a face-to-face π-π interaction with the NAM. The mGlu_2_ and mGlu_3_ 7TM binding pockets are identical apart from the three residues (mGlu_2_/mGlu_3_): Ile/Val^5×51^, Asn/Asp^5×47^ and His/Val723^45×52^. The first, Ile/Val^5×51^, merely lines the bottom of the binding site (Fig. 1c,d); consequently it is not expected to be a major determinant for selectivity.

In mGlu_2_, Asn735^5×47^ is 3.9 Å from the amide nitrogen in our mGlu_2_-RO5488608 binding mode analysis (Fig. 1c), and the effect is subtle upon mutation to the corresponding mGlu_3_ residue, aspartate, consistent with a non-optimal weak interaction that can also be formed with aspartate. This is obviously not the case for several mGlu_2_ PAMs, where potentiation is largely affected by the same mutation^39^. In contrast, mGlu_2_ to mGlu_3_ conversion of the third position, i.e. H723^45×52^V mutagenesis, yielded a large effect on RO5488608 potency^29^. This is consistent with our proposed binding mode in which His723^45×52^ forms π-π interactions to the RO5488608 bi-phenyl, as well as a hydrogen bond to the sulphonic acid (Fig. 1c,d), neither of which can be formed by valine. Furthermore, His723^45×52^ is unique for mGlu_2_ and may therefore offer selectivity against the other seven mGlu subtypes.

In our mGlu_3_-ML337 binding model the NAM interacts with ECL2 by van der Waals contact to Val732^45×52^ and hydrogen bonds to the backbone (Fig. 1d). This could be sufficient to explain the selectivity, as the corresponding mGlu_2_ residue is a histidine that occupies much of this site, and is likely to prevent the hydrogen bonds. The other two residues that differ compared to mGlu_2_, Asp744^5×47^ and Val748^5×51^, do not seem to interact with ML337 and in our mGlu_3_ model Asp744^5×47^ is predicted to be neutral and thus present the same interaction possibilities as the corresponding asparagine of mGlu_2_. Though Val732^45×52^ is unique to mGlu_3_ (Table 1), the corresponding threonine in group I mGlu receptors, and possibly also the methionine of mGlu_6_, would also allow for the accommodation of this ligand. Hence, the mGlu_3_ binding pocket features three residues; Phe632^2×56^, Phe652^3×40^ and Val732^45×52^, representing opportunities for selective ligand interactions.

#### Group III selectivity hotspots

Our comparison of the mGlu 7TM domain pockets pinpointed only the residue, Ser5×47, which may be utilized to gain group III selectivity (Figs 2 and 3). This residue forms a hydrogen bond in our selected MMPIP-mGlu_7_ binding mode (Fig. 1e). Another alternative binding pose of MMPIP displayed no specific interaction to Ser763^5×47^, which is why this was not selected. The other mGlu groups contain asparagines/aspartate in this position that could also form hydrogen bonds, but their side chains are longer and could block group III selective modulators or abolish/weaken the interaction.

In mGlu_7_, there are three unique residues: Gln755^5×39^, Ile756^5×40^ and Val800^6×49^ (Fig. 2). The latter, Val800^6×49^ is located at the border of the binding site and is not that different from the isoleucine in mGlu_6_ and mGlu_8_, making it unlikely that this residue could yield selectivity. Ile756^5×40^ is more promising and displays favourable van der Waals contacts in the MMPIP-mGlu_7_ binding mode analysis (Fig. 1e). The other three group III subtypes contain a leucine in position 5×40, which in our binding model would cause steric clashes with MMPIP in the most frequent (~95%) rotamers. Gln755^5×39^ also displays van der Waals contacts with the selected MMPIP binding pose (to the methoxy moiety), but in accordance with the available SAR information^21^ we do not observe a direct hydrogen bond. All other mGlu subtypes contain a serine or glycine in position 5×39. Consequently, Gln755^5×39^ and Ile756^5×40^ offer clear opportunities for selectivity, whereas only long ligands would be able to reach Val800^6×49^.

The remaining group III receptors lack selective NAMs and have very few unique residues. In mGlu_4_, there is one unique binding pocket residue, Leu756^5×43^, and though its location at the border of the binding site in-between TM3 and 5 renders it difficult to reach mutation to Ser/Lys has been shown to affect the function of one PAM^40^. Thus, information from mGlu_4_ selective PAMs^12^ may also indicate how to obtain mGlu_4_ selective NAMs. In mGlu_6_, Thr661^3×40^ is unique and could contribute to selectivity, whereas mGlu_8_ lacks unique residues. Thus, the rational design of NAMs with selectivity for the individual group III members is very challenging, however selectivity could potentially still be achieved by exploiting a combination of several residues.

### The structural basis of NAM/PAM molecular switches

**Figure 4:**
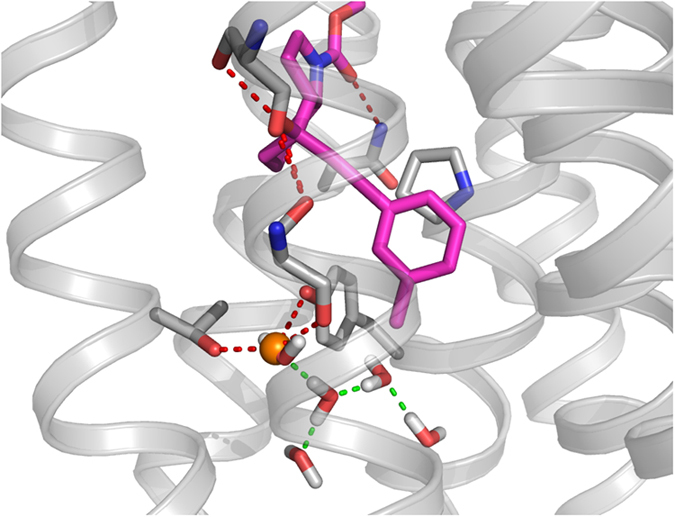
The structural basis of NAM/PAM molecular switches. A crystallographic water molecule in the mGlu_5_ mavoglurant binding site (mavoglurant in magenta, water molecule in orange) hydrogen bonds to Tyr659^3x44^, Thr781^6x46^, and the main-chain of Ser809^7x40^. A predicted network of waters is shown overlaid with h-bonds (green). This network of water molecules is proposed to facilitate activation. Subtle changes to allosteric modulators influence the stability of this water network, and may lead to molecular switching within closely related series of molecules.

In the field of mGlu NAM/PAM discovery, mode-switching has been observed as a result of mutations in different positions in the allosteric binding site, e.g. 3×44^41^, 6×50^36^ and 6×53^34^,^42^ but also in several diverse chemical series^43^,^44^ as a result of small structural changes to the modulator. This phenomenon has also been reported for numerous mavoglurant-related alkyne linker series^45^,^46^,^47^,^48^ making the recent mGlu_5_-mavoglurant structure an ideal template in which to further investigate this observation. The NAM/PAM switch mechanism of mavoglurant-related compounds seems to be through occupancy of the mGlu_5_ specific sub-pocket and in the mGlu_5_ crystal structure a water molecule coordinated to Tyr659^3×44^, Thr781^6×46^, and the main-chain of Ser809^7×40^ is located directly adjacent to the 3-methyl substituent of mavoglurant^32^ (Fig. 4). This water is calculated to be very stable using WaterFLAP software^49^ and further prediction of water molecules in the apo-mGlu_5_ cavity places a water molecule 0.5 Å from the position observed in the crystal structure (Fig. 4). This predicted network of water molecules could move to fill voids, and create different hydrogen bonding networks, therefore lowering the energy required to reach the active state. Subtle variations of mGlu_5_ ligands can change the environment the water network feels, thereby stabilizing or destabilizing the water molecules and lowering or raising the barrier to activation. This is borne out by changes in the hydrophobicity of this region, e.g. mutation of Thr781^6×46^ and Ser809^7×40^ to alanine switches the pharmacology of alkyne PAMs to NAMs^26^.

## Discussion

The release of crystal structures for class A GPCRs has repeatedly been followed by new high-affinity ligands identified by docking-based virtual screening^50^,^51^ – also for models of related subtypes^52^,^53^. The non-crystallized mGlu_2–4_ and mGlu_6–8_ subtypes all have high sequence similarity to mGlu_5_: 67–71% and 68–74% within the 7TM domain and binding pocket, respectively (Supplementary Table 5). Still, the subtype differences, as described herein, necessitate careful optimization of the binding sites residues to allow for favourable ligand interactions and, conversely, to distance blocking residues. Also the prospects of wider class C GPCR modeling outside of the mGlu family have increased considerably; and mGlu_1_ and mGlu_5_ display sequence similarities to other class C GPCRs of 39% or more for the 7TM domain and 35% or more for the binding site residues (Supplementary Table 6). All models should be refined by factoring in the accumulated mutagenesis and SAR data, and validated by docking of known modulator series, where available. As the mGlu receptors in particular, and additional class C GPCRs, are highly pursued by academia and industry, it is expected that we will see examples of new modulators in the near future.

For some subtypes, like mGlu_4_, the main therapeutic interest lies in PAMs as potential treatments for e.g. Parkinson’s disease^12^, and it is an intriguing question whether the available NAM-bound mGlu receptor crystal structures can be utilized for discovery of PAMs. The 7TM backbone show only moderate movements around the ligand binding pocket in class A GPCR crystal structures of active and inactive states^54^. However, the rotamers within a binding site are tightly linked to the pharmacological activity and this biases structure-based virtual screening towards the same ligand activities and scaffolds. Thus, the application of the current mGlu structures in PAM discovery, should start by re-optimization of the binding site side chains around high-affinity PAMs^53^.

Unintended NAM/PAM mode-switching can also occur presenting obstacles to drug design, for example variation of the 3-methyl substituent from methoxy to chloro to fluoro in benzaldazine compounds switches the ligand from a NAM to a neutral binder to a PAM, respectively^43^. We suggest that ligand induced changes to a water network in mGlu_5_ (Fig. 4) may constitute the mechanism of such a switch. However, though the residues interacting with the water molecule observed in the mGlu_5_ crystal structure are conserved across the mGlu family (Fig. 2), this switch mechanism of mavoglurant-related compounds seems to be through occupancy of the mGlu_5_ specific sub-pocket and thus may only be relevant to mGlu_5_. However, it is apparent from mutations, also in mGlu_1_, that different positions in the allosteric site can determine the effect of a modulator^34^,^36^,^41^,^42^ and should be considered in the development of mGlu NAMs for other subtypes as well. These changes are in most cases difficult to predict, but further structural biology and structure-activity studies could serve to map mechanisms switching negative and positive modulation.

In rhodopsin, the tryptophan of the highly conserved FxxCWxP motif in TM6 is denoted a “toggle switch” and proposed to adopt alternative rotameric states upon activation^55^. The mGlu receptors all contain a tryptophan in the equivalent position of TM6 (6×50), but in the mGlu crystal structures Trp6×50 adopts a different rotamer compared to e.g. rhodopsin and displays hydrogen bonds to TM5. Whilst this rotameric state of Trp6×50 cannot currently be implicated in mGlu activation, it is critical for the NAM binding modes observed in both mGlu structures. However, assuming that Trp6×50 is a “toggle switch” in the mGlu receptors imply a different rotamer in the active state receptor conformation and thus a differently shaped PAM binding site, which could be relevant to consider in PAM discovery and development.

**Figure 5:**
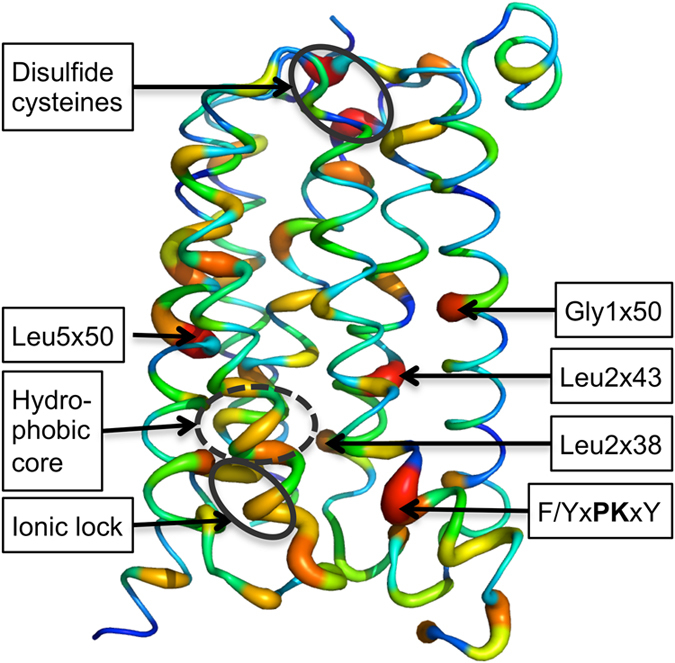
Class C sequence identity mapped onto mGlu5 crystal structure. The mGlu_5_ structure shown in putty representation with sequence identity conservation of Class C vertebrates mapped onto the structure. The sequence identity conservation is based on the alignment presented by Dore et al.^32^ and the size of helix bulge and color (blue to red) indicates where there is greater sequence identity conservation.

Whilst the class C and A GPCR sequences are quite dissimilar, many of the structural microdomains known to stabilize the receptor active/inactive states are in topographically similar positions (Fig. 5)^32^. On the intracellular, G protein binding side, an “ionic lock” connects the highly conserved Lys3×50 and Glu6×35 (one turn higher than in class A GPCRs); and alanine mutations of these residues significantly increase basal activity^32^. Furthermore, the F/YxPKxY motif in the intracellular end of TM7 in class C has been proposed to interact with the highly conserved Lys7×51 and/or Phe/Tyr7×48 to stabilize the inter-helical space created by the outward movement of TM6, seen in the fully activated Class A receptor structures. The Phe/Tyr7×48 part of this motif appears to be stabilized in the inactive form via interactions with Leu2×43 and Phe1×61. Another region that has been shown in Class A to affect basal activity levels is the hydrophobic core of the receptor^56^ and the equivalent positions in class C, 3×43, 6×39 and 6×42, have conserved physiochemical characteristics. Furthermore, Leu2×38 is in part playing the role of stabilizing TM3 in place for the Class C receptors. Other residues implicated, Leu5×50 and Tyr5×46 in TM5 also appear structurally significant in stabilizing TM3 within the helical bundle. Tyr5×46 is additionally bridging TM5 to TM4, where the hydroxyl group of Tyr5×46 interacts with the backbone carbonyl of Leu4×39 in TM4, breaking the helical hydrogen bonding pattern and influencing the trajectory of the extracellular portion of TM4. In summary, a greater understanding of Class C structure and function would further be very valuable to be able to readily design modulators that can stabilize the desired receptor state.

In conclusion, the new mGlu receptor crystal structures offer unprecedented drug discovery opportunities, as well as a new understanding of the molecular microdomains and switches underlying receptor function. Mutagenesis and SAR data can now be mapped to the structures to explain modulator actions, and the herein presented analysis combines these data presenting a base for rational structure-based drug design. The comparative binding mode/site studies show that several selectivity hotspots exist for group I–II, but few for group III, mGlu receptors. Still, more data is needed, in particular structures of PAM-mGlu receptor complexes and the first selective NAMs for mGlu_3_, mGlu_4_ and mGlu_6–8_ would serve to expand the current set of tools, and help to elucidate the therapeutic implications of the individual subtypes. Taken together, it can be expected that the current crystal structures are only the start of an increased activity in mGlu, as well as class C GPCR drug design expected to spawn many new potent and selective modulators.

## Methods

### Homology modeling

Homology models of mGlu_1_, mGlu_2_, mGlu_3_, mGlu_5_ and mGlu_7_ were constructed using Modeller^57^, version 9.13. Both the mGlu_1_^9^ and mGlu_5_^32^ crystal structures downloaded from the Protein Data Bank^58^ (PDB IDs: 4OR2 and 4OO9) were used as templates for the mGlu_2_, mGlu_3_ and mGlu_7_ models, while the mGlu_1_ model where modeled on the mGlu_5_ structure and vice versa. The templates were aligned to the target sequences using Clustal X^59^, version 2.1. Protein sequences were retrieved from UniProt (www.uniprot.org) with the following accession codes: Q13255 (mGlu_1_), Q14416 (mGlu_2_), Q14832 (mGlu_3_), P41594 (mGlu_5_) and Q14831 (mGlu_7_). In order to retain the side chain conformations of residues in the binding site conserved between the target sequences and the template structures we disabled the randomization of the starting structure by using the “a.very_fast()” Modeller keyword. This results in a single model that is only subjected to a brief optimization. This procedure was selected based on the high sequence similarities and assumed structural conservation combined with the fact that the binding site residues are optimized relative to the ligand in following computational steps. The homology models were prepared for docking with the Protein Preparation Wizard (2014-2; Epik, version 2.4; Impact, version 5.9; Prime, version 3.2, Shrödinger, LLC, New York, NY, 2011) using default settings.

### Induced fit docking

The chemical structures of the NAMs, FITM, RO5488608, ML337, mavoglurant and MMPIP were downloaded from the PubChem database (https://pubchem.ncbi.nlm.nih.gov, CIDs: 53233900, 73755206, 60204017, 9926832 and 9945530, respectively), imported into Maestro (version 9.8, Schrödinger, LLC, New York, NY, 2014) and subjected to a conformational search in MacroModel (version 10.4, Schrödinger, LLC, New York, NY, 2014) using the MCMM search method and automatic setup. For FITM, ML337, mavoglurant and MMPIP only the lowest energy conformation was used as input for the docking, but for RO5488608 there are two low energy conformations of the 7-membered heterocycle, both of which were used. A pyridine analogue of FITM was build from the lowest energy conformation of FITM in Maestro by altering the aromatic carbon atom in the 2-position of the benzamide to a nitrogen atom.

FITM and the analogue, RO5488608, ML337, mavoglurant and MMPIP were docked into their respective mGlu receptor subtypes (mGlu_1_, mGlu_2_, mGlu_3_, mGlu_5_ and mGlu_7_, respectively) using the induced-fit docking protocol in the Schrödinger Molecular Modeling Suite (2014-2, Glide version 6.1, Prime version 3.4, Schrödinger, LLC, New York, NY, 2014). Residues with long/bulky or very different side chains not conserved in the templates and/or that sterically block part of the binding site occupied by FITM or mavoglurant in the mGlu crystal structures are mutated to alanine (mGlu_1_: T7×33, mGlu_2_ and mGlu_3_: L3×36 and M7×33, mGlu_5_: none, mGlu_7_: L3×36, M3×40 and M7×33). The receptor grid for docking was calculated with the centre defined by residue positions 3×32, 3×33, 3×44, 6×50 and 6×53 and no scaling of van der Waals radii. Ligand docking was performed with standard precision and 70% van der Waals radii scaling of all ligand atoms. The top 20 scoring ligand poses are optimized in complex with the receptor by first re-introducing and sampling the side chains of the mutated residues and energy minimizing the ligand and all residues within 5 Å. A final step of re-docking is applied with ligand van der Waals scaling of 80%.

Among the top ten ranked ligand complexes we compared the ligand binding modes and discarded the lowest ranking poses with heavy atom RMSD values below 0.8 Å relative to other poses. Next we considered the ligand conformational energy, the number of hydrogen bonds and steric clashes to the protein. For RO5488608 we used the available mutational data and considered the vicinity of the poses to the residues known to affect the inhibitory action of RO5488608. For ML337 and MMPIP the sparse SAR information available was also taken into account. For MMPIP the above criteria did not allow for a clear selection between two possible binding poses and thus we utilized the fact that MMPIP is known to be mGlu_7_ selective and included interactions to subtype specific residues as a final selection criterion. Among the unique binding poses for each docked ligand, RO5488608, ML337 and MMPIP ranked 4^th^, 3^rd^ and 1^st^ but a detailed description of the binding pose selection is available in Supplementary Tables 2–4 and corresponding table legends.

### Binding site characterization

Binding site characterization was performed on the mGlu_1_ and mGlu_5_ crystal structures and the mGlu_2_, mGlu_3_ and mGlu_7_ homology models after induced-fit docking using SiteMap (version 3.1, Schrödinger, LLC, New York, NY, 2014) with default settings. In all cases the NAM binding site ranked first according to both SiteScore and Drugability Score. For visualization purposes of the size and shape of the binding site (Fig. 1 and Supplementary Fig. 1) we displayed the SitePoints from SiteMap as a surface with van der Waals radius of 1.0 Å.

### Water modeling in mGlu_5_

Prediction and placement of water molecules in mGlu_5_ was performed with WaterFLAP^49^ using the mGlu_5_ crystal structure without the ligand and water molecules.

## Additional Information

**How to cite this article**: Harpsøe, K. *et al.* Selective Negative Allosteric Modulation Of Metabotropic Glutamate Receptors – A Structural Perspective of Ligands and Mutants. *Sci. Rep.* **5**, 13869; doi: 10.1038/srep13869 (2015).

## Supplementary Material

Supplementary Information

Supplementary Dataset 1

## Figures and Tables

**Table 1:** mGlu receptor therapeutic areas and prototypic allosteric modulators.

**Group**	**Subtype**	**Therapeutic Area**	**Prototypic NAMs**	**Prototypic PAMs**
I	mGlu_1_	**NAMs:** Fragile X syndrome, anxiety, Alzheimer’s disease, schizophrenia, pain and addiction^1^,^15^,^16^	FITM, CPCCOEt	Ro67-7476
	mGlu_5_	**NAMs:** Fragile X syndrome, anxiety, chronic pain, depression, migraine, Parkinson’s disease, gastroesophageal reflux disease, epilepsy and addiction^1^,^4^,^5^,^13^,^14^,^15^,^16^	MPEP, Mavoglurant, Fenobam	CPPHA, CDPPB
		**PAMs:** Anxiety, Huntington’s disease, schizophrenia^1^,^4^,^5^,^15^,^16^		
II	mGlu_2_	**PAMs:** Addiction, Alzheimer’s disease, anxiety, depression, pain, schizophrenia^1^,^4^,^5^,^15^,^16^	RO5488608	BINA, LY487379
	mGlu_3_	**NAMs:** Depression^1^,^16^	ML337	—
		**PAMs:** Alzheimer’s disease, pain, anxiety^1^		
III	mGlu_4_	**PAMs:** Schizophrenia, pain, multiple sclerosis, Parkinson’s disease^4^,^5^,^11^,^12^,^15^,^16^	—	PHCCC
	mGlu_6_	Congenital stationary night blindness^1^,^5^	—	—
	mGlu_7_	**NAMs:** depression, anxiety^1^,^15^,^16^,^20^	MMPIP	AMN082
	mGlu_8_		—	—
