# Supplementary material for: Selective Negative Allosteric Modulation Of Metabotropic Glutamate Receptors – A Structural Perspective of Ligands and Mutants
Source: Sci Rep. 2015 Sep 11;5:13869. doi: 10.1038/srep13869 (PMC4566082; doi:10.1038/srep13869)
Supplement: Supplementary Information [file srep13869-s1.pdf]

Selective Negative Allosteric Modulation Of Metabotropic Glutamate Receptors – A Structural Perspective of Ligands and Mutants

Kasper Harpsøe[1], Vignir Isberg[1], Benjamin G Tehan[2], Dahlia Weiss[2], Angela Arsova[1], Fiona H. Marshall[2], Hans Bräuner-Osborne[1] and David Gloriam[1]

[1]Department of Drug Design and Pharmacology, Faculty of Health and Medical Sciences, University of Copenhagen, Jagtvej 162, 2100 Copenhagen, Denmark; and [2]Heptares Therapeutics Ltd, BioPark, Broadwater Road, Welwyn Garden City, AL7 3AX, UK

Supplementary Information

List of Contents

Supplementary Figure 1: FITM and mavoglurant poses in crystal structure complexes and docking in homology models. 2

Supplementary Figure 2: The unique sub-pocket of $mGlu_5$ and blocking residues of other mGlu receptors........ 3

Supplementary Figure 3: Docking pose of a pyridine analogue of FITM 4

Supplementary Table 1: mGlu receptor mutants and their effects 5

Supplementary Table 2: Docking pose selection for RO5488608 35

Supplementary Table 3: Docking pose selection of ML337 36

Supplementary Table 4: Docking pose selection of MMPIP 37

Supplementary Table 5: Similarity matrix of the mGlu receptors based on the 7TM domain and the binding site residues 38

Supplementary Table 6: Similarity between the mGlu crystal structures and other selected class C GPCRs........ 38

References 39

Supplementary Figure 1: FITM and mavoglurant poses in crystal structure complexes and docking in homology models.

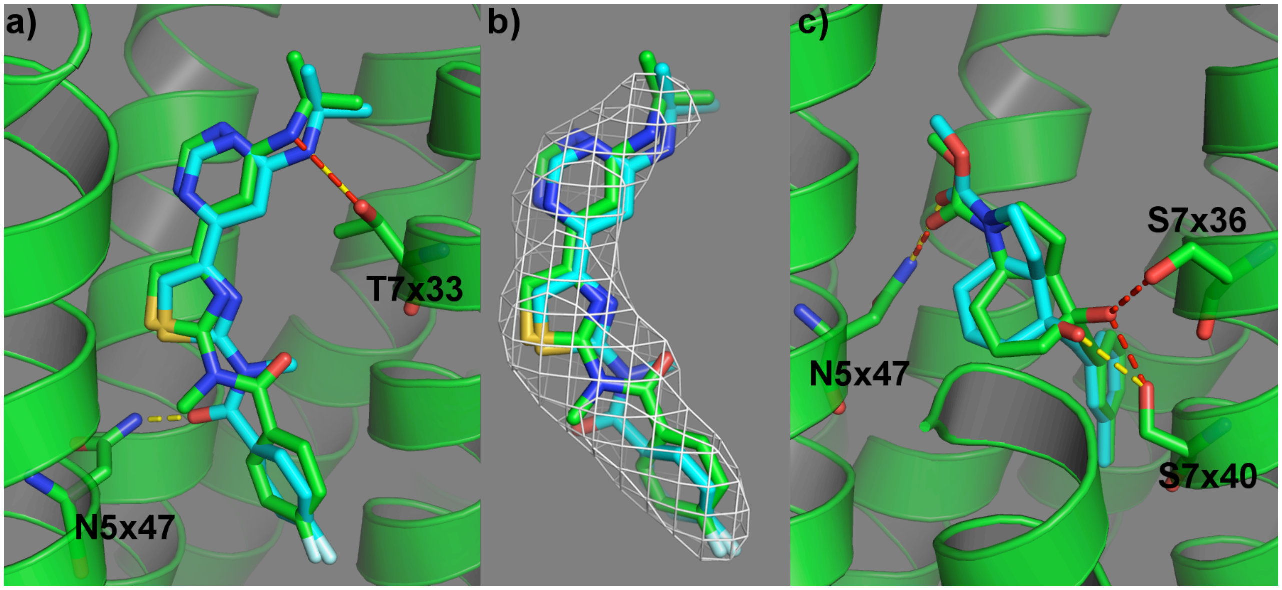

Docking FITM and mavoglurant into homology models of mGlu$_1$ and mGlu$_5$, respectively, results in binding poses that closely match those observed in the crystal structures. An mGlu$_1$ homology model was build using the mGlu$_5$ crystal structure as template and vice versa. FITM and mavoglurant were docked into their respective receptor models using an induced fit docking protocol. Comparing the best ranking docking poses (cyan carbon atoms) of, **A)**, FITM in the mGlu$_1$ model and, **C)**, mavoglurant in the mGlu$_5$ model to the binding modes in the crystal structures (green carbon atoms) by superimposing the proteins result in RMSD values on all ligand heavy atoms of 1.83 and 0.67 Å, respectively. **A)** In our docking of FITM, the *N*-Methyl amide moiety is flipped horizontally, and we observe a hydrogen bond (yellow dashed lines) to Asn5x47, which is not present in the crystal structure (red dashed lines). **B)** Comparing the two different binding poses to the crystal structure electron density at an isovalue of 1 (white grid) it is apparent that both are within the density and thus indistinguishable in spite of the 1.83 Å RMSD. **C)** For mavoglurant we observe a small shift in the binding mode resulting in loss of a hydrogen bond to Ser7x40 compared to the hydrogen bonding pattern in the crystal structure (yellow and red dashed lines, respectively).

Supplementary Figure 2: The unique sub-pocket of $mGlu_5$ and blocking residues of other mGlu receptors

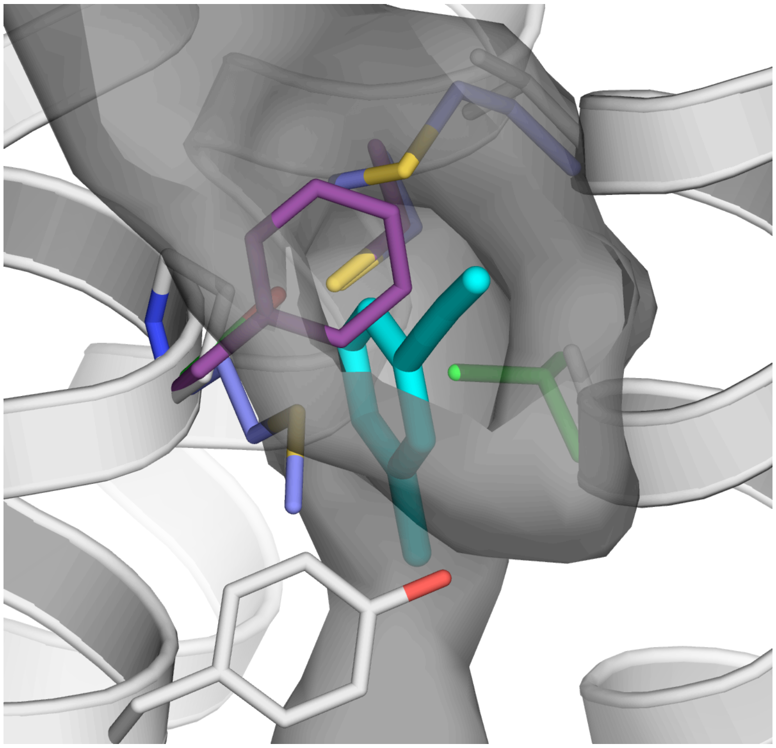

Comparative binding cavity analysis shows that the deep mavoglurant sub-pocket is unique for $mGlu_5$, as all other subtypes contain blocking side chains. The surface of the sub-pocket deep in-between TM2, 3 and 7 (grey) in the $mGlu_5$ crystal structure (white cartoon and sticks) with the 2-methylacetylene moiety of mavoglurant (cyan sticks). Shown as thin sticks are the residues that block this sub-pocket in the mGlu1 crystal structure (green) plus the $mGlu_2$ (magenta) model as representative of group II and $mGlu_7$ model (purple) as representative of group III.

Supplementary Figure 3: Docking pose of a pyridine analogue of FITM

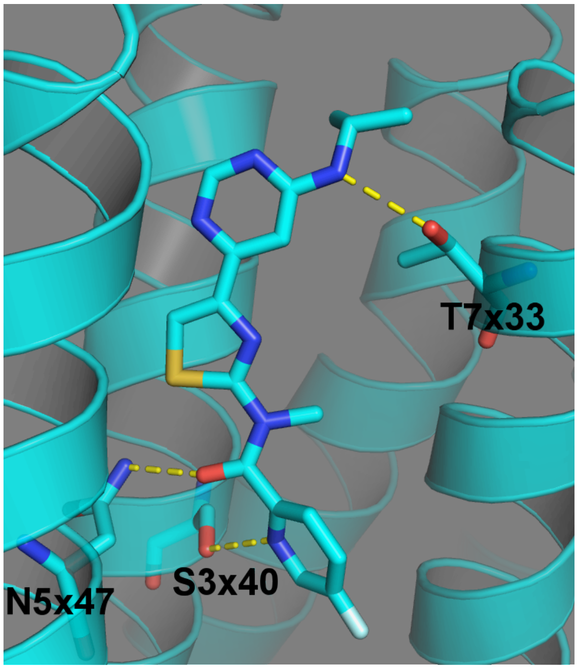

Docking the pyridine analogue of FITM results in an additional hydrogen bond to Ser3x40. The top ranked induced fit docking pose of a pyridine analogue of FITM in $mGlu_1$ shows a binding mode identical to that observed for FITM (**Supplementary Figure 1**) with hydrogen bonds (yellow dashed lines) to Asn5x47 and Thr7x33. An additional hydrogen bond from the pyridine nitrogen to the $mGlu_1$ specific Ser3x40 could result in even larger $mGlu_1$ selectivity than observed for the parent compound, FITM.

Supplementary Table 1: mGlu receptor mutants and their effects

[a] Numbers refer to either the human, (h) or rat (r) mGlu sequence, [b] Modulator class is in parentheses; (N) for NAMs and (P) for PAMs, [c] Effect on NAM/PAM function (inhibition/potentiation in different kinds of assays) is italic and given as *IC/EC_{50}(mutant)/IC/EC_{50}(wild-type)*; effect on NAM/PAM binding affinity is bold and given as **$K_{D/I/B}$(mutant)/$K_{D/I/B}$(wild-type)**; When a fold-change could not be calculated a qualitative description is given, e.g. abolished potentiation. ECL1-2 denotes that a residue is in a structurally non-conserved part of extracellular loop 1-3.

Subtype	GPCRdb	Mutation[a]	Modulator[b]	Effect (Fold)[c]	Ref.
$mGlu_1$	1x46	F599I (r)	CPPHA (P)	*Abolished potentiation*	1
$mGlu_1$	1x46	F599I (r)	Ro 67-7476 (P)	*No significant effect*	1
$mGlu_1$	3x32	Q660A (h)	FITM (N)	**1.0**	2
$mGlu_1$	3x36	V664I (h)	FITM (N)	**0.99**	2
$mGlu_1$	3x40	S668P (h)	FITM (N)	**2.1**	2
$mGlu_1$	3x41	A669V (r)	[^{3}H]EM-TBPC (N)	**0.45**	3
$mGlu_1$	3x44	Y672F (r)	[^{3}H]EM-TBPC (N)	**1.3**	3
$mGlu_1$	3x44	Y672V (r)	[^{3}H]EM-TBPC (N)	**4.8**	3
$mGlu_1$	4x37	S711F (h)	CFMMC (N)	*0.65*	4
$mGlu_1$	4x41	S715C (h)	CFMMC (N)	*0.65*	4
$mGlu_1$	4x42	V716I (h)	CFMMC (N)	*0.37*	4
$mGlu_1$	4x45	T719G (h)	CFMMC (N)	*0.53*	4
$mGlu_1$	4x49	T723A (r)	[^{3}H]EM-TBPC (N)	**0.92**	3
$mGlu_1$	4x49	T723A (h)	CFMMC (N)	*0.53*	4
$mGlu_1$	4x51	I725F (h)	CFMMC (N)	*10.2*	4
$mGlu_1$	4x51	I725F (h)	Compound 1 (N)	*0.65*	4
$mGlu_1$	4x51	I725F (h)	FTIDC (N)	*0.42*	4
$mGlu_1$	4x51	I725F (h)	JNJ16259685 (N)	*0.49*	4
$mGlu_1$	4x51	I725F (h)	LY456066 (N)	*0.58*	4
$mGlu_1$	4x51	I725F (h)	YM-298198 (N)	*0.68*	4
$mGlu_1$	45x51	N747A (r)	[^{3}H]EM-TBPC (N)	**1.2**	3
$mGlu_1$	45x51	N747A (r)	EM-TBPC (N)	*0.24*	3
$mGlu_1$	45x52	T748A (h)	FITM (N)	**1.9**	2
$mGlu_1$	45x53	S749A (r)	[^{3}H]EM-TBPC (N)	**1.5**	3
$mGlu_1$	45x53	S749T (r)	[^{3}H]EM-TBPC (N)	**1.3**	3
$mGlu_1$	5x37	N750A (r)	[^{3}H]EM-TBPC (N)	**1.0**	3
$mGlu_1$	5x37	N750Q (r)	[^{3}H]EM-TBPC (N)	**1.0**	3
$mGlu_1$	5x37	N750A (r)	EM-TBPC (N)	*0.08*	3
$mGlu_1$	5x42	A755T (h)	CFMMC (N)	*0.44*	4

$mGlu_1$	5x43	P756S (h)	FITM (N)	**2.6**	2
$mGlu_1$	5x44	L757V (h)	$[^3H]$EM-TBPC (N)	**Gain of binding** (EM-TBPC does not bind to WT human mGlu1)	3
$mGlu_1$	5x44	V757A (r)	$[^3H]$EM-TBPC (N)	**12.9**	3
$mGlu_1$	5x44	V757L (r)	$[^3H]$EM-TBPC (N)	**12.7**	3
$mGlu_1$	5x44	V757L (r)	CPPHA (P)	*No significant effect*	1
$mGlu_1$	5x44	V757L (r)	EM-TBPC (N)	*9.1*	3
$mGlu_1$	5x44	L757V (h)	Ro 67-7476 (P)	*Gain of potentiation (does not potentiate WT human mGlu1)*	5
$mGlu_1$	5x44	V757L (r)	Ro 67-7476 (P)	*Abolished potentiation*	1
$mGlu_1$	5x44	V757L (r)	Ro 67-7476 (P)	*Abolished potentiation*	6
$mGlu_1$	5x44	V757L (r)	Ro 67-7476 (P)	*Abolished potentiation*	5
$mGlu_1$	5x44	V757L (r)	VU-48 (P)	*Abolished potentiation*	6
$mGlu_1$	5x44	V757L (r)	VU-71 (P)	*Abolished potentiation*	6
$mGlu_1$	5x47	N760A (h)	CFMMC (N)	*>67*	4
$mGlu_1$	5x47	N760A (h)	Compound 1 (N)	*8.1*	4
$mGlu_1$	5x47	N760A (h)	FTIDC (N)	*0.7*	4
$mGlu_1$	5x47	N760A (h)	JNJ16259685 (N)	*1.3*	4
$mGlu_1$	5x47	N760A (h)	LY456066 (N)	*0.16*	4
$mGlu_1$	5x47	N760A (h)	YM-298198 (N)	*5.4*	4
$mGlu_1$	5x52	M765L (h)	CFMMC (N)	*0.64*	4
$mGlu_1$	5x56	Y769F (h)	CFMMC (N)	*0.54*	4
$mGlu_1$	6x46	T794A (h)	FITM (N)	**0.61**	2
$mGlu_1$	6x50	W798F (r)	$[^3H]$EM-TBPC (N)	**0.11**	3
$mGlu_1$	6x50	W798Y (r)	$[^3H]$EM-TBPC (N)	**0.20**	3
$mGlu_1$	6x50	W798A (h)	CFMMC (N)	*0.25*	4
$mGlu_1$	6x50	W798A (h)	Compound 1 (N)	*0.27*	4
$mGlu_1$	6x50	W798F (r)	EM-TBPC (N)	*0.03*	3
$mGlu_1$	6x50	W798A (h)	FTIDC (N)	*0.63*	4
$mGlu_1$	6x50	W798A (h)	FTIDC (N)	*0.8*	7
$mGlu_1$	6x50	W798A (h)	JNJ16259685 (N)	*0.79*	4
$mGlu_1$	6x50	W798A (h)	LY456066 (N)	*2.6*	4
$mGlu_1$	6x50	W798A (h)	YM-298198 (N)	*0.56*	4
$mGlu_1$	6x53	F801A (r)	$[^3H]$EM-TBPC (N)	**Abolished binding**	3
$mGlu_1$	6x53	F801A (h)	CFMMC (N)	*>140*	4
$mGlu_1$	6x53	F801A (h)	Compound 1 (N)	*380*	4
$mGlu_1$	6x53	F801A (h)	FTIDC (N)	*67*	4
$mGlu_1$	6x53	F801A (h)	FTIDC (N)	*90*	7
$mGlu_1$	6x53	F801A (h)	JNJ16259685 (N)	*>2700*	4
$mGlu_1$	6x53	F801A (h)	LY456066 (N)	*0.20*	4
$mGlu_1$	6x53	F801A (h)	YM-298198 (N)	*NAM to PAM switch*	4
$mGlu_1$	6x54	V802M (r)	$[^3H]$EM-TBPC (N)	**0.42**	3
$mGlu_1$	6x57	Y805A (r)	$[^3H]$EM-TBPC (N)	**Abolished binding**	3

$mGlu_1$	6x57	Y805A (h)	CFMMC	(N)	*>200*	4
$mGlu_1$	6x57	Y805A (h)	Compound 1	(N)	*0.44*	4
$mGlu_1$	6x57	Y805A (r)	EM-TBPC	(N)	*>78*	3
$mGlu_1$	6x57	Y805A (h)	FTIDC	(N)	*0.84*	4
$mGlu_1$	6x57	Y805A (h)	FTIDC	(N)	*1.4*	7
$mGlu_1$	6x57	Y805A (h)	JNJ16259685	(N)	*13*	4
$mGlu_1$	6x57	Y805A (h)	LY456066	(N)	*9.1*	4
$mGlu_1$	6x57	Y805A (h)	YM-298198	(N)	*12*	4
$mGlu_1$	7x33	T815M (r)	[^{3}H]EM-TBPC	(N)	**Abolished binding**	3
$mGlu_1$	7x33	T815M (h)	CFMMC	(N)	*>200*	4
$mGlu_1$	7x33	T815M (h)	Compound 1	(N)	*4.2*	4
$mGlu_1$	7x33	T815M (r)	EM-TBPC	(N)	*>78*	3
$mGlu_1$	7x33	T815A (h)	FITM	(N)	**2.8**	2
$mGlu_1$	7x33	T815M (h)	FITM	(N)	**5.7**	2
$mGlu_1$	7x33	T815M (h)	FTIDC	(N)	*38*	4
$mGlu_1$	7x33	T815M (h)	FTIDC	(N)	*72*	7
$mGlu_1$	7x33	T815M (h)	JNJ16259685	(N)	*>2700*	4
$mGlu_1$	7x33	T815M (h)	LY456066	(N)	*>350*	4
$mGlu_1$	7x33	T815M (h)	YM-298198	(N)	*>110*	4
$mGlu_1$	7x36	A818I (r)	[^{3}H]EM-TBPC	(N)	**1.3**	3
$mGlu_1$	7x36	A818S (r)	[^{3}H]EM-TBPC	(N)	**0.80**	3
$mGlu_1$	7x36	A818S (r)	EM-TBPC	(N)	*0.79*	3
$mGlu_1$	7x36	A818S (h)	FITM	(N)	**0.65**	2
$mGlu_1$	7x40	S822A (h)	FITM	(N)	**0.80**	2
$mGlu_1$	7x41	V823A (h)	[^{3}H]M-MPEP	(N)	**No gain of binding** (MPEP does not bind to human WT $mGlu_1$)	8
$mGlu_2$	2x49	C616S (h)	BINA	(P)	*No significant effect*	9
$mGlu_2$	2x49	C616S (h)	JNJ-35814376	(P)	*No significant effect*	9
$mGlu_2$	2x49	C616S (h)	JNJ-40068782	(P)	*No significant effect*	9
$mGlu_2$	2x49	C616S (h)	JNJ-40297036	(P)	*No significant effect*	9
$mGlu_2$	2x49	C616S (h)	JNJ-41482012	(P)	*No significant effect*	9
$mGlu_2$	2x49	C616S (h)	JNJ-42329001	(P)	*No significant effect*	9
$mGlu_2$	2x49	C616S (h)	JNJ-46281222	(P)	*No significant effect*	9
$mGlu_2$	2x49	C616S (h)	LY2607540	(P)	*No significant effect*	9
$mGlu_2$	2x49	C616S (h)	LY487379	(P)	*No significant effect*	9
$mGlu_2$	2x49	C616S (r)	RO4988546	(N)	*1.0*	10
$mGlu_2$	2x49	C616S (r)	RO5488608	(N)	*0.8*	10
$mGlu_2$	2x50	Y617F (r)	RO4988546	(N)	*1.3*	10
$mGlu_2$	2x50	Y617F (r)	RO5488608	(N)	*1.1*	10
$mGlu_2$	2x53	T620A (r)	RO4988546	(N)	*0.8*	10
$mGlu_2$	2x53	T620A (r)	RO5488608	(N)	*1.0*	10
$mGlu_2$	2x55	I622F (h)	BINA	(P)	*No significant effect*	9
$mGlu_2$	2x55	I622F (h)	JNJ-35814376	(P)	*No significant effect*	9

$mGlu_2$	2x55	I622F (h)	JNJ-40068782	(P)	*No significant effect*	9
$mGlu_2$	2x55	I622F (h)	JNJ-40297036	(P)	*No significant effect*	9
$mGlu_2$	2x55	I622F (h)	JNJ-41482012	(P)	*No significant effect*	9
$mGlu_2$	2x55	I622F (h)	JNJ-42329001	(P)	*No significant effect*	9
$mGlu_2$	2x55	I622F (h)	JNJ-46281222	(P)	*No significant effect*	9
$mGlu_2$	2x55	I622F (h)	LY2607540	(P)	*No significant effect*	9
$mGlu_2$	2x55	I622F (h)	LY487379	(P)	*No significant effect*	9
$mGlu_2$	3x32	R635A (h)	BINA	(P)	*6.6*	9
$mGlu_2$	3x32	R635A (h)	JNJ-35814376	(P)	*1.3*	9
$mGlu_2$	3x32	R635A (h)	JNJ-40068782	(P)	*3.0*	9
$mGlu_2$	3x32	R635A (h)	JNJ-40297036	(P)	*No significant effect*	9
$mGlu_2$	3x32	R635A (h)	JNJ-41482012	(P)	*No significant effect*	9
$mGlu_2$	3x32	R635A (h)	JNJ-42329001	(P)	*No significant effect*	9
$mGlu_2$	3x32	R635A (h)	JNJ-46281222	(P)	*No significant effect*	9
$mGlu_2$	3x32	R635A (h)	LY2607540	(P)	*No significant effect*	9
$mGlu_2$	3x32	R635A (h)	LY487379	(P)	*No significant effect*	9
$mGlu_2$	3x32	R635A (r)	RO4988546	(N)	*15.2, 18.0*	10
$mGlu_2$	3x32	R635A (r)	RO5488608	(N)	*2.3, 38.3*	10
$mGlu_2$	3x33	R636A (h)	BINA	(P)	*No significant effect*	9
$mGlu_2$	3x33	R636A (h)	JNJ-35814376	(P)	*No significant effect*	9
$mGlu_2$	3x33	R636A (h)	JNJ-40068782	(P)	*No significant effect*	9
$mGlu_2$	3x33	R636A (h)	JNJ-40297036	(P)	*No significant effect*	9
$mGlu_2$	3x33	R636A (h)	JNJ-41482012	(P)	*No significant effect*	9
$mGlu_2$	3x33	R636A (h)	JNJ-42329001	(P)	*No significant effect*	9
$mGlu_2$	3x33	R636A (h)	JNJ-46281222	(P)	*No significant effect*	9
$mGlu_2$	3x33	R636A (h)	LY2607540	(P)	*No significant effect*	9
$mGlu_2$	3x33	R636A (h)	LY487379	(P)	*No significant effect*	9
$mGlu_2$	3x33	R636A (r)	RO4988546	(N)	*4.1, 21.8*	10
$mGlu_2$	3x33	R636A (r)	RO5488608	(N)	*>100, 36.0*	10
$mGlu_2$	3x36	L639A (h)	BINA	(P)	*14.8*	9
$mGlu_2$	3x36	L639A (h)	JNJ-35814376	(P)	*2.3*	9
$mGlu_2$	3x36	L639A (h)	JNJ-40068782	(P)	*4.3*	9
$mGlu_2$	3x36	L639A (h)	JNJ-40297036	(P)	*3.2*	9
$mGlu_2$	3x36	L639A (h)	JNJ-41482012	(P)	*No significant effect*	9
$mGlu_2$	3x36	L639A (h)	JNJ-42329001	(P)	*No significant effect*	9
$mGlu_2$	3x36	L639A (h)	JNJ-46281222	(P)	*No significant effect*	9
$mGlu_2$	3x36	L639A (h)	LY2607540	(P)	*1.6*	9
$mGlu_2$	3x36	L639A (h)	LY487379	(P)	*4.2*	9
$mGlu_2$	3x36	L639A (r)	RO4988546	(N)	*1.2*	10
$mGlu_2$	3x36	L639A (r)	RO5488608	(N)	*1.7*	10
$mGlu_2$	3x38	T641S (h)	BINA	(P)	*No significant effect*	9
$mGlu_2$	3x38	T641S (h)	JNJ-35814376	(P)	*No significant effect*	9
$mGlu_2$	3x38	T641S (h)	JNJ-40068782	(P)	*No significant effect*	9

mGlu$_2$	3x38	T641S (h)	JNJ-40297036	(P)	*No significant effect*	[9]
mGlu$_2$	3x38	T641S (h)	JNJ-41482012	(P)	*No significant effect*	[9]
mGlu$_2$	3x38	T641S (h)	JNJ-42329001	(P)	*No significant effect*	[9]
mGlu$_2$	3x38	T641S (h)	JNJ-46281222	(P)	*No significant effect*	[9]
mGlu$_2$	3x38	T641S (h)	LY2607540	(P)	*No significant effect*	[9]
mGlu$_2$	3x38	T641S (h)	LY487379	(P)	*No significant effect*	[9]
mGlu$_2$	3x39	A642S (h)	BINA	(P)	*No significant effect*	[9]
mGlu$_2$	3x39	A642S (h)	JNJ-35814376	(P)	*No significant effect*	[9]
mGlu$_2$	3x39	A642S (h)	JNJ-40068782	(P)	*No significant effect*	[9]
mGlu$_2$	3x39	A642S (h)	JNJ-40297036	(P)	*No significant effect*	[9]
mGlu$_2$	3x39	A642S (h)	JNJ-41482012	(P)	*No significant effect*	[9]
mGlu$_2$	3x39	A642S (h)	JNJ-42329001	(P)	*No significant effect*	[9]
mGlu$_2$	3x39	A642S (h)	JNJ-46281222	(P)	*No significant effect*	[9]
mGlu$_2$	3x39	A642S (h)	LY2607540	(P)	*No significant effect*	[9]
mGlu$_2$	3x39	A642S (h)	LY487379	(P)	*No significant effect*	[9]
mGlu$_2$	3x39	A642S (r)	RO4988546	(N)	*1.1*	[10]
mGlu$_2$	3x39	A642S (r)	RO5488608	(N)	*1.6*	[10]
mGlu$_2$	3x40	F643A (h)	BINA	(P)	*15.1*	[9]
mGlu$_2$	3x40	F643A (h)	JNJ-35814376	(P)	*No significant effect*	[9]
mGlu$_2$	3x40	F643A (h)	JNJ-40068782	(P)	*18.2*	[9]
mGlu$_2$	3x40	F643A (h)	JNJ-40297036	(P)	*>61.7*	[9]
mGlu$_2$	3x40	F643A (h)	JNJ-41482012	(P)	*>55*	[9]
mGlu$_2$	3x40	F643A (h)	JNJ-42329001	(P)	*147.9*	[9]
mGlu$_2$	3x40	F643A (h)	JNJ-46281222	(P)	*58.9*	[9]
mGlu$_2$	3x40	F643A (h)	LY2607540	(P)	*7.8*	[9]
mGlu$_2$	3x40	F643A (h)	LY487379	(P)	*>60.3*	[9]
mGlu$_2$	3x40	F643A (r)	RO4988546	(N)	*19.3, 5.9*	[10]
mGlu$_2$	3x40	F643A (r)	RO5488608	(N)	*1.2, 3.4*	[10]
mGlu$_2$	3x41	S644A (h)	BINA	(P)	*No significant effect*	[9]
mGlu$_2$	3x41	S644A (h)	JNJ-35814376	(P)	*No significant effect*	[9]
mGlu$_2$	3x41	S644A (h)	JNJ-40068782	(P)	*No significant effect*	[9]
mGlu$_2$	3x41	S644A (h)	JNJ-40297036	(P)	*No significant effect*	[9]
mGlu$_2$	3x41	S644A (h)	JNJ-41482012	(P)	*No significant effect*	[9]
mGlu$_2$	3x41	S644A (h)	JNJ-42329001	(P)	*No significant effect*	[9]
mGlu$_2$	3x41	S644A (h)	JNJ-46281222	(P)	*No significant effect*	[9]
mGlu$_2$	3x41	S644A (h)	LY2607540	(P)	*No significant effect*	[9]
mGlu$_2$	3x41	S644A (h)	LY487379	(P)	*No significant effect*	[9]
mGlu$_2$	3x41	S644A (r)	RO4988546	(N)	*0.9*	[10]
mGlu$_2$	3x41	S644A (r)	RO5488608	(N)	*1.1*	[10]
mGlu$_2$	3x44	Y647V (r)	RO4988546	(N)	*2.9, 3.4*	[10]
mGlu$_2$	3x44	Y647V (r)	RO5488608	(N)	*0.8, 1.8*	[10]
mGlu$_2$	4x34	A681F (h)	BINA	(P)	*No significant effect*	[9]
mGlu$_2$	4x34	A681F (h)	JNJ-35814376	(P)	*No significant effect*	[9]

$mGlu_2$	4x34	A681F (h)	JNJ-40068782	(P)	*No significant effect*	9
$mGlu_2$	4x34	A681F (h)	JNJ-40297036	(P)	*No significant effect*	9
$mGlu_2$	4x34	A681F (h)	JNJ-41482012	(P)	*No significant effect*	9
$mGlu_2$	4x34	A681F (h)	JNJ-42329001	(P)	*No significant effect*	9
$mGlu_2$	4x34	A681F (h)	JNJ-46281222	(P)	*No significant effect*	9
$mGlu_2$	4x34	A681F (h)	LY2607540	(P)	*No significant effect*	9
$mGlu_2$	4x34	A681F (h)	LY487379	(P)	*No significant effect*	9
$mGlu_2$	4x34	A681F (h)	LY487379	(P)	*No significant effect*	11
$mGlu_2$	4x34	A681F (h)	MRLSD-650	(P)	*No significant effect*	12
$mGlu_2$	4x41	S688L (h)	BINA	(P)	*1.5*	9
$mGlu_2$	4x41	S688L (h)	JNJ-35814376	(P)	*1.2*	9
$mGlu_2$	4x41	S688L (h)	JNJ-40068782	(P)	*1.4*	9
$mGlu_2$	4x41	S688L (h)	JNJ-40297036	(P)	*0.89*	9
$mGlu_2$	4x41	S688L (h)	JNJ-41482012	(P)	*0.87*	9
$mGlu_2$	4x41	S688L (h)	JNJ-42329001	(P)	*1.1*	9
$mGlu_2$	4x41	S688L (h)	JNJ-46281222	(P)	*3.4*	9
$mGlu_2$	4x41	S688L (h)	LY2607540	(P)	*1.1*	9
$mGlu_2$	4x41	S688L (h)	LY487379	(P)	*0.85*	9
$mGlu_2$	4x41	S688L (h)	LY487379	(P)	*No significant effect*	11
$mGlu_2$	4x41	S688L (h)	MRLSD-650	(P)	*No significant effect*	12
$mGlu_2$	4x42	G689V (h)	BINA	(P)	*6.8*	9
$mGlu_2$	4x42	G689V (h)	JNJ-35814376	(P)	*2.8*	9
$mGlu_2$	4x42	G689V (h)	JNJ-40068782	(P)	*4.3*	9
$mGlu_2$	4x42	G689V (h)	JNJ-40297036	(P)	*3.1*	9
$mGlu_2$	4x42	G689V (h)	JNJ-41482012	(P)	*>55*	9
$mGlu_2$	4x42	G689V (h)	JNJ-42329001	(P)	*3.7*	9
$mGlu_2$	4x42	G689V (h)	JNJ-46281222	(P)	*7.6*	9
$mGlu_2$	4x42	G689V (h)	LY2607540	(P)	*5.6*	9
$mGlu_2$	4x42	G689V (h)	LY487379	(P)	*1.6*	9
$mGlu_2$	4x42	G689V (h)	LY487379	(P)	*No significant effect*	11
$mGlu_2$	4x42	G689V (h)	MRLSD-650	(P)	*No significant effect*	12
$mGlu_2$	4x46	I693M (h)	BINA	(P)	*No significant effect*	9
$mGlu_2$	4x46	I693M (h)	JNJ-35814376	(P)	*No significant effect*	9
$mGlu_2$	4x46	I693M (h)	JNJ-40068782	(P)	*No significant effect*	9
$mGlu_2$	4x46	I693M (h)	JNJ-40297036	(P)	*No significant effect*	9
$mGlu_2$	4x46	I693M (h)	JNJ-41482012	(P)	*No significant effect*	9
$mGlu_2$	4x46	I693M (h)	JNJ-42329001	(P)	*No significant effect*	9
$mGlu_2$	4x46	I693M (h)	JNJ-46281222	(P)	*No significant effect*	9
$mGlu_2$	4x46	I693M (h)	LY2607540	(P)	*No significant effect*	9
$mGlu_2$	4x46	I693M (h)	LY487379	(P)	*No significant effect*	9
$mGlu_2$	4x48	V695S (h)	BINA	(P)	*No significant effect*	9
$mGlu_2$	4x48	V695S (h)	JNJ-35814376	(P)	*No significant effect*	9
$mGlu_2$	4x48	V695S (h)	JNJ-40068782	(P)	*No significant effect*	9

$mGlu_2$	4x48	V695S (h)	JNJ-40297036	(P)	*No significant effect*	9
$mGlu_2$	4x48	V695S (h)	JNJ-41482012	(P)	*No significant effect*	9
$mGlu_2$	4x48	V695S (h)	JNJ-42329001	(P)	*No significant effect*	9
$mGlu_2$	4x48	V695S (h)	JNJ-46281222	(P)	*No significant effect*	9
$mGlu_2$	4x48	V695S (h)	LY2607540	(P)	*No significant effect*	9
$mGlu_2$	4x48	V695S (h)	LY487379	(P)	*No significant effect*	9
$mGlu_2$	4x49	A696V (h)	BINA	(P)	*No significant effect*	9
$mGlu_2$	4x49	A696V (h)	JNJ-35814376	(P)	*No significant effect*	9
$mGlu_2$	4x49	A696V (h)	JNJ-40068782	(P)	*No significant effect*	9
$mGlu_2$	4x49	A696V (h)	JNJ-40297036	(P)	*No significant effect*	9
$mGlu_2$	4x49	A696V (h)	JNJ-41482012	(P)	*No significant effect*	9
$mGlu_2$	4x49	A696V (h)	JNJ-42329001	(P)	*No significant effect*	9
$mGlu_2$	4x49	A696V (h)	JNJ-46281222	(P)	*No significant effect*	9
$mGlu_2$	4x49	A696V (h)	LY2607540	(P)	*No significant effect*	9
$mGlu_2$	4x49	A696V (h)	LY487379	(P)	*No significant effect*	9
$mGlu_2$	4x53	V700L (h)	BINA	(P)	*No significant effect*	9
$mGlu_2$	4x53	V700L (h)	JNJ-35814376	(P)	*No significant effect*	9
$mGlu_2$	4x53	V700L (h)	JNJ-40068782	(P)	*No significant effect*	9
$mGlu_2$	4x53	V700L (h)	JNJ-40297036	(P)	*No significant effect*	9
$mGlu_2$	4x53	V700L (h)	JNJ-41482012	(P)	*No significant effect*	9
$mGlu_2$	4x53	V700L (h)	JNJ-42329001	(P)	*No significant effect*	9
$mGlu_2$	4x53	V700L (h)	JNJ-46281222	(P)	*No significant effect*	9
$mGlu_2$	4x53	V700L (h)	LY2607540	(P)	*No significant effect*	9
$mGlu_2$	4x53	V700L (h)	LY487379	(P)	*No significant effect*	9
$mGlu_2$	ECL2	G706R (h)	BINA	(P)	*No significant effect*	9
$mGlu_2$	ECL2	G706R (h)	JNJ-35814376	(P)	*No significant effect*	9
$mGlu_2$	ECL2	G706R (h)	JNJ-40068782	(P)	*No significant effect*	9
$mGlu_2$	ECL2	G706R (h)	JNJ-40297036	(P)	*No significant effect*	9
$mGlu_2$	ECL2	G706R (h)	JNJ-41482012	(P)	*No significant effect*	9
$mGlu_2$	ECL2	G706R (h)	JNJ-42329001	(P)	*No significant effect*	9
$mGlu_2$	ECL2	G706R (h)	JNJ-46281222	(P)	*No significant effect*	9
$mGlu_2$	ECL2	G706R (h)	LY2607540	(P)	*No significant effect*	9
$mGlu_2$	ECL2	G706R (h)	LY487379	(P)	*No significant effect*	9
$mGlu_2$	ECL2	E708Y (h)	BINA	(P)	*No significant effect*	9
$mGlu_2$	ECL2	E708Y (h)	JNJ-35814376	(P)	*No significant effect*	9
$mGlu_2$	ECL2	E708Y (h)	JNJ-40068782	(P)	*No significant effect*	9
$mGlu_2$	ECL2	E708Y (h)	JNJ-40297036	(P)	*No significant effect*	9
$mGlu_2$	ECL2	E708Y (h)	JNJ-41482012	(P)	*No significant effect*	9
$mGlu_2$	ECL2	E708Y (h)	JNJ-42329001	(P)	*No significant effect*	9
$mGlu_2$	ECL2	E708Y (h)	JNJ-46281222	(P)	*No significant effect*	9
$mGlu_2$	ECL2	E708Y (h)	LY2607540	(P)	*No significant effect*	9
$mGlu_2$	ECL2	E708Y (h)	LY487379	(P)	*No significant effect*	9
$mGlu_2$	ECL2	A710L (h)	BINA	(P)	*No significant effect*	9

$mGlu_2$	ECL2	A710L (h)	JNJ-35814376	(P)	*No significant effect*	9
$mGlu_2$	ECL2	A710L (h)	JNJ-40068782	(P)	*No significant effect*	9
$mGlu_2$	ECL2	A710L (h)	JNJ-40297036	(P)	*No significant effect*	9
$mGlu_2$	ECL2	A710L (h)	JNJ-41482012	(P)	*No significant effect*	9
$mGlu_2$	ECL2	A710L (h)	JNJ-42329001	(P)	*No significant effect*	9
$mGlu_2$	ECL2	A710L (h)	JNJ-46281222	(P)	*No significant effect*	9
$mGlu_2$	ECL2	A710L (h)	LY2607540	(P)	*No significant effect*	9
$mGlu_2$	ECL2	A710L (h)	LY487379	(P)	*No significant effect*	9
$mGlu_2$	ECL2	P711A (h)	BINA	(P)	*No significant effect*	9
$mGlu_2$	ECL2	P711A (h)	JNJ-35814376	(P)	*No significant effect*	9
$mGlu_2$	ECL2	P711A (h)	JNJ-40068782	(P)	*No significant effect*	9
$mGlu_2$	ECL2	P711A (h)	JNJ-40297036	(P)	*No significant effect*	9
$mGlu_2$	ECL2	P711A (h)	JNJ-41482012	(P)	*No significant effect*	9
$mGlu_2$	ECL2	P711A (h)	JNJ-42329001	(P)	*No significant effect*	9
$mGlu_2$	ECL2	P711A (h)	JNJ-46281222	(P)	*No significant effect*	9
$mGlu_2$	ECL2	P711A (h)	LY2607540	(P)	*No significant effect*	9
$mGlu_2$	ECL2	P711A (h)	LY487379	(P)	*No significant effect*	9
$mGlu_2$	ECL2	V716T (h)	BINA	(P)	*No significant effect*	9
$mGlu_2$	ECL2	V716T (h)	JNJ-35814376	(P)	*No significant effect*	9
$mGlu_2$	ECL2	V716T (h)	JNJ-40068782	(P)	*No significant effect*	9
$mGlu_2$	ECL2	V716T (h)	JNJ-40297036	(P)	*No significant effect*	9
$mGlu_2$	ECL2	V716T (h)	JNJ-41482012	(P)	*No significant effect*	9
$mGlu_2$	ECL2	V716T (h)	JNJ-42329001	(P)	*No significant effect*	9
$mGlu_2$	ECL2	V716T (h)	JNJ-46281222	(P)	*No significant effect*	9
$mGlu_2$	ECL2	V716T (h)	LY2607540	(P)	*No significant effect*	9
$mGlu_2$	ECL2	V716T (h)	LY487379	(P)	*No significant effect*	9
$mGlu_2$	ECL2	T718I (h)	BINA	(P)	*No significant effect*	9
$mGlu_2$	ECL2	T718I (h)	JNJ-35814376	(P)	*No significant effect*	9
$mGlu_2$	ECL2	T718I (h)	JNJ-40068782	(P)	*No significant effect*	9
$mGlu_2$	ECL2	T718I (h)	JNJ-40297036	(P)	*No significant effect*	9
$mGlu_2$	ECL2	T718I (h)	JNJ-41482012	(P)	*No significant effect*	9
$mGlu_2$	ECL2	T718I (h)	JNJ-42329001	(P)	*No significant effect*	9
$mGlu_2$	ECL2	T718I (h)	JNJ-46281222	(P)	*No significant effect*	9
$mGlu_2$	ECL2	T718I (h)	LY2607540	(P)	*No significant effect*	9
$mGlu_2$	ECL2	T718I (h)	LY487379	(P)	*No significant effect*	9
$mGlu_2$	45x52	H723V (h)	BINA	(P)	*No significant effect*	9
$mGlu_2$	45x52	H723V (h)	JNJ-35814376	(P)	*2.5*	9
$mGlu_2$	45x52	H723V (h)	JNJ-40068782	(P)	*4.3*	9
$mGlu_2$	45x52	H723V (h)	JNJ-40297036	(P)	*2.1*	9
$mGlu_2$	45x52	H723V (h)	JNJ-41482012	(P)	*1.1*	9
$mGlu_2$	45x52	H723V (h)	JNJ-42329001	(P)	*No significant effect*	9
$mGlu_2$	45x52	H723V (h)	JNJ-46281222	(P)	*No significant effect*	9
$mGlu_2$	45x52	H723V (h)	LY2607540	(P)	*2.6*	9

$mGlu_2$	45x52	H723V (h)	LY487379	(P)	*No significant effect*	[9]
$mGlu_2$	45x52	H723V (h)	LY487379	(P)	*No significant effect*	[11]
$mGlu_2$	45x52	H723V (h)	MRLSD-650	(P)	*No significant effect*	[12]
$mGlu_2$	45x52	H723F (r)	RO4988546	(N)	*2.9, 3.5*	[10]
$mGlu_2$	45x52	H723V (r)	RO4988546	(N)	*8.2, 6.9*	[10]
$mGlu_2$	45x52	H723F (r)	RO5488608	(N)	*28.3, 45.9*	[10]
$mGlu_2$	45x52	H723V (r)	RO5488608	(N)	*8.2, 52.0*	[10]
$mGlu_2$	5x37	D725A (h)	BINA	(P)	*No significant effect*	[9]
$mGlu_2$	5x37	D725A (h)	JNJ-35814376	(P)	*No significant effect*	[9]
$mGlu_2$	5x37	D725A (h)	JNJ-40068782	(P)	*No significant effect*	[9]
$mGlu_2$	5x37	D725A (h)	JNJ-40297036	(P)	*No significant effect*	[9]
$mGlu_2$	5x37	D725A (h)	JNJ-41482012	(P)	*No significant effect*	[9]
$mGlu_2$	5x37	D725A (h)	JNJ-42329001	(P)	*No significant effect*	[9]
$mGlu_2$	5x37	D725A (h)	JNJ-46281222	(P)	*No significant effect*	[9]
$mGlu_2$	5x37	D725A (h)	LY2607540	(P)	*No significant effect*	[9]
$mGlu_2$	5x37	D725A (h)	LY487379	(P)	*No significant effect*	[9]
$mGlu_2$	5x38	A726S (h)	BINA	(P)	*No significant effect*	[9]
$mGlu_2$	5x38	A726S (h)	JNJ-35814376	(P)	*No significant effect*	[9]
$mGlu_2$	5x38	A726S (h)	JNJ-40068782	(P)	*No significant effect*	[9]
$mGlu_2$	5x38	A726S (h)	JNJ-40297036	(P)	*No significant effect*	[9]
$mGlu_2$	5x38	A726S (h)	JNJ-41482012	(P)	*No significant effect*	[9]
$mGlu_2$	5x38	A726S (h)	JNJ-42329001	(P)	*No significant effect*	[9]
$mGlu_2$	5x38	A726S (h)	JNJ-46281222	(P)	*No significant effect*	[9]
$mGlu_2$	5x38	A726S (h)	LY2607540	(P)	*No significant effect*	[9]
$mGlu_2$	5x38	A726S (h)	LY487379	(P)	*No significant effect*	[9]
$mGlu_2$	5x40	M728A (h)	BINA	(P)	*No significant effect*	[9]
$mGlu_2$	5x40	M728A (h)	JNJ-35814376	(P)	*No significant effect*	[9]
$mGlu_2$	5x40	M728A (h)	JNJ-40068782	(P)	*No significant effect*	[9]
$mGlu_2$	5x40	M728A (h)	JNJ-40297036	(P)	*No significant effect*	[9]
$mGlu_2$	5x40	M728A (h)	JNJ-41482012	(P)	*No significant effect*	[9]
$mGlu_2$	5x40	M728A (h)	JNJ-42329001	(P)	*No significant effect*	[9]
$mGlu_2$	5x40	M728A (h)	JNJ-46281222	(P)	*No significant effect*	[9]
$mGlu_2$	5x40	M728A (h)	LY2607540	(P)	*No significant effect*	[9]
$mGlu_2$	5x40	M728A (h)	LY487379	(P)	*No significant effect*	[9]
$mGlu_2$	5x40	M728A (r)	RO4988546	(N)	*0.3, 0.2*	[10]
$mGlu_2$	5x40	M728A (r)	RO5488608	(N)	*0.3, 0.9*	[10]
$mGlu_2$	5x42	G730I (h)	BINA	(P)	*No significant effect*	[9]
$mGlu_2$	5x42	G730I (h)	JNJ-35814376	(P)	*No significant effect*	[9]
$mGlu_2$	5x42	G730I (h)	JNJ-40068782	(P)	*No significant effect*	[9]
$mGlu_2$	5x42	G730I (h)	JNJ-40297036	(P)	*No significant effect*	[9]
$mGlu_2$	5x42	G730I (h)	JNJ-41482012	(P)	*No significant effect*	[9]
$mGlu_2$	5x42	G730I (h)	JNJ-42329001	(P)	*No significant effect*	[9]
$mGlu_2$	5x42	G730I (h)	JNJ-46281222	(P)	*No significant effect*	[9]

$mGlu_2$	5x42	G730I (h)	LY2607540	(P)	*No significant effect*	[9]
$mGlu_2$	5x42	G730I (h)	LY487379	(P)	*No significant effect*	[9]
$mGlu_2$	5x42	G730I (h)	LY487379	(P)	*No significant effect*	[11]
$mGlu_2$	5x42	G730I (h)	MRLSD-650	(P)	*No significant effect*	[12]
$mGlu_2$	5x43	S731A (h)	BINA	(P)	*No significant effect*	[9]
$mGlu_2$	5x43	S731A (h)	JNJ-35814376	(P)	*1.9*	[9]
$mGlu_2$	5x43	S731A (h)	JNJ-40068782	(P)	*No significant effect*	[9]
$mGlu_2$	5x43	S731A (h)	JNJ-40297036	(P)	*No significant effect*	[9]
$mGlu_2$	5x43	S731A (h)	JNJ-41482012	(P)	*No significant effect*	[9]
$mGlu_2$	5x43	S731A (h)	JNJ-42329001	(P)	*No significant effect*	[9]
$mGlu_2$	5x43	S731A (h)	JNJ-46281222	(P)	*No significant effect*	[9]
$mGlu_2$	5x43	S731A (h)	LY2607540	(P)	*2.6*	[9]
$mGlu_2$	5x43	S731A (h)	LY487379	(P)	*5.0*	[9]
$mGlu_2$	5x43	S731A (r)	RO4988546	(N)	*0.7, 0.1*	[10]
$mGlu_2$	5x43	S731A (r)	RO5488608	(N)	*0.9, 1.5*	[10]
$mGlu_2$	5x44	L732A (h)	BINA	(P)	*0.17*	[9]
$mGlu_2$	5x44	L732A (h)	JNJ-35814376	(P)	*0.93*	[9]
$mGlu_2$	5x44	L732A (h)	JNJ-40068782	(P)	*0.7*	[9]
$mGlu_2$	5x44	L732A (h)	JNJ-40297036	(P)	*1.9*	[9]
$mGlu_2$	5x44	L732A (h)	JNJ-41482012	(P)	*>55*	[9]
$mGlu_2$	5x44	L732A (h)	JNJ-42329001	(P)	*14.5*	[9]
$mGlu_2$	5x44	L732A (h)	JNJ-46281222	(P)	*6.0*	[9]
$mGlu_2$	5x44	L732A (h)	LY2607540	(P)	*3.0*	[9]
$mGlu_2$	5x44	L732A (h)	LY487379	(P)	*>60.3*	[9]
$mGlu_2$	5x44	L732A (r)	RO4988546	(N)	*9.1, 2.8*	[10]
$mGlu_2$	5x44	L732A (r)	RO5488608	(N)	*30.6, 40.3*	[10]
$mGlu_2$	5x45	A733T (h)	BINA	(P)	*No significant effect*	[9]
$mGlu_2$	5x45	A733T (h)	JNJ-35814376	(P)	*No significant effect*	[9]
$mGlu_2$	5x45	A733T (h)	JNJ-40068782	(P)	*No significant effect*	[9]
$mGlu_2$	5x45	A733T (h)	JNJ-40297036	(P)	*No significant effect*	[9]
$mGlu_2$	5x45	A733T (h)	JNJ-41482012	(P)	*No significant effect*	[9]
$mGlu_2$	5x45	A733T (h)	JNJ-42329001	(P)	*No significant effect*	[9]
$mGlu_2$	5x45	A733T (h)	JNJ-46281222	(P)	*No significant effect*	[9]
$mGlu_2$	5x45	A733T (h)	LY2607540	(P)	*No significant effect*	[9]
$mGlu_2$	5x45	A733T (h)	LY487379	(P)	*No significant effect*	[9]
$mGlu_2$	5x45	A733T (h)	LY487379	(P)	*No significant effect*	[11]
$mGlu_2$	5x45	A733T (h)	MRLSD-650	(P)	*No significant effect*	[12]
$mGlu_2$	5x47	N735D (h)	BINA	(P)	*79.4*	[9]
$mGlu_2$	5x47	N735D (h)	BINA	(P)	*Abolished potentiation*	[13]
$mGlu_2$	5x47	N735D (h)	JNJ-35814376	(P)	*>15.5*	[9]
$mGlu_2$	5x47	N735D (h)	JNJ-40068782	(P)	*24.0*	[9]
$mGlu_2$	5x47	N735D (h)	JNJ-40297036	(P)	*>61.7*	[9]
$mGlu_2$	5x47	N735D (h)	JNJ-41482012	(P)	*>55*	[9]

mGlu$_2$	5x47	N735D (h)	JNJ-42329001 (P)	*27.5*	9
mGlu$_2$	5x47	N735D (h)	JNJ-46281222 (P)	*26.3*	9
mGlu$_2$	5x47	N735D (h)	LY2607540 (P)	*27.5*	9
mGlu$_2$	5x47	N735D (h)	LY487379 (P)	*>60.3*	9
mGlu$_2$	5x47	N735D (h)	LY487379 (P)	*Reduced potentiation*	11
mGlu$_2$	5x47	N735D (h)	LY487379 (P)	*Reduced potentiation*	13
mGlu$_2$	5x47	N735D (h)	MNI-135 (N)	*No significant effect*	13
mGlu$_2$	5x47	N735D (h)	MNI-136 (N)	*No significant effect*	13
mGlu$_2$	5x47	N735D (h)	MNI-137 (N)	*No significant effect*	13
mGlu$_2$	5x47	N735D (h)	MRLSD-650 (P)	*Reduced potentiation*	12
mGlu$_2$	5x47	N735D (r)	RO4988546 (N)	*1.4, 4.3*	10
mGlu$_2$	5x47	N735D (r)	RO5488608 (N)	*1.3, 10.4*	10
mGlu$_2$	5x48	V736A (h)	BINA (P)	*No significant effect*	9
mGlu$_2$	5x48	V736A (h)	JNJ-35814376 (P)	*No significant effect*	9
mGlu$_2$	5x48	V736A (h)	JNJ-40068782 (P)	*No significant effect*	9
mGlu$_2$	5x48	V736A (h)	JNJ-40297036 (P)	*No significant effect*	9
mGlu$_2$	5x48	V736A (h)	JNJ-41482012 (P)	*No significant effect*	9
mGlu$_2$	5x48	V736A (h)	JNJ-42329001 (P)	*No significant effect*	9
mGlu$_2$	5x48	V736A (h)	JNJ-46281222 (P)	*No significant effect*	9
mGlu$_2$	5x48	V736A (h)	LY2607540 (P)	*No significant effect*	9
mGlu$_2$	5x48	V736A (h)	LY487379 (P)	*No significant effect*	9
mGlu$_2$	5x48	V736A (r)	RO4988546 (N)	*0.5, 0.6*	10
mGlu$_2$	5x48	V736A (r)	RO5488608 (N)	*0.5, 1.9*	10
mGlu$_2$	5x52	A740I (h)	BINA (P)	*No significant effect*	9
mGlu$_2$	5x52	A740I (h)	JNJ-35814376 (P)	*No significant effect*	9
mGlu$_2$	5x52	A740I (h)	JNJ-40068782 (P)	*No significant effect*	9
mGlu$_2$	5x52	A740I (h)	JNJ-40297036 (P)	*No significant effect*	9
mGlu$_2$	5x52	A740I (h)	JNJ-41482012 (P)	*No significant effect*	9
mGlu$_2$	5x52	A740I (h)	JNJ-42329001 (P)	*No significant effect*	9
mGlu$_2$	5x52	A740I (h)	JNJ-46281222 (P)	*No significant effect*	9
mGlu$_2$	5x52	A740I (h)	LY2607540 (P)	*No significant effect*	9
mGlu$_2$	5x52	A740I (h)	LY487379 (P)	*No significant effect*	9
mGlu$_2$	5x52	A740I (h)	LY487379 (P)	*No significant effect*	11
mGlu$_2$	5x52	A740I (h)	MRLSD-650 (P)	*No significant effect*	12
mGlu$_2$	6x46	T769S (r)	RO4988546 (N)	*0.4*	10
mGlu$_2$	6x46	T769V (r)	RO4988546 (N)	*0.5*	10
mGlu$_2$	6x46	T769S (r)	RO5488608 (N)	*0.9*	10
mGlu$_2$	6x46	T769V (r)	RO5488608 (N)	*0.8*	10
mGlu$_2$	6x50	W773A (h)	BINA (P)	*107.2*	9
mGlu$_2$	6x50	W773A (h)	JNJ-35814376 (P)	*>15.5*	9
mGlu$_2$	6x50	W773A (h)	JNJ-40068782 (P)	*>79.4*	9
mGlu$_2$	6x50	W773A (h)	JNJ-40297036 (P)	*>61.7*	9
mGlu$_2$	6x50	W773A (h)	JNJ-41482012 (P)	*>55*	9

$mGlu_2$	6x50	W773A (h)	JNJ-42329001 (P)	*32.4*	9
$mGlu_2$	6x50	W773A (h)	JNJ-46281222 (P)	*9.6*	9
$mGlu_2$	6x50	W773A (h)	LY2607540 (P)	*93.3*	9
$mGlu_2$	6x50	W773A (h)	LY487379 (P)	*>60.3*	9
$mGlu_2$	6x50	W773A (r)	RO4988546 (N)	*178.9, 0.2*	10
$mGlu_2$	6x50	W773F (r)	RO4988546 (N)	*11.4, 6.8*	10
$mGlu_2$	6x50	W773A (r)	RO5488608 (N)	*Abolished inhibition, 2.1*	10
$mGlu_2$	6x50	W773F (r)	RO5488608 (N)	*1.9, 3.8*	10
$mGlu_2$	6x53	F776A (h)	BINA (P)	*2.1*	9
$mGlu_2$	6x53	F776A (h)	JNJ-35814376 (P)	*No significant effect*	9
$mGlu_2$	6x53	F776A (h)	JNJ-40068782 (P)	*3.3*	9
$mGlu_2$	6x53	F776A (h)	JNJ-40297036 (P)	*No significant effect*	9
$mGlu_2$	6x53	F776A (h)	JNJ-41482012 (P)	*No significant effect*	9
$mGlu_2$	6x53	F776A (h)	JNJ-42329001 (P)	*1.8*	9
$mGlu_2$	6x53	F776A (h)	JNJ-46281222 (P)	*No significant effect*	9
$mGlu_2$	6x53	F776A (h)	LY2607540 (P)	*1.1*	9
$mGlu_2$	6x53	F776A (h)	LY487379 (P)	*>60.3*	9
$mGlu_2$	6x54	L777A (r)	RO4988546 (N)	*0.9*	10
$mGlu_2$	6x54	L777A (r)	RO5488608 (N)	*1.0*	10
$mGlu_2$	6x57	F780A (h)	BINA (P)	*No significant effect*	9
$mGlu_2$	6x57	F780A (h)	JNJ-35814376 (P)	*No significant effect*	9
$mGlu_2$	6x57	F780A (h)	JNJ-40068782 (P)	*No significant effect*	9
$mGlu_2$	6x57	F780A (h)	JNJ-40297036 (P)	*No significant effect*	9
$mGlu_2$	6x57	F780A (h)	JNJ-41482012 (P)	*No significant effect*	9
$mGlu_2$	6x57	F780A (h)	JNJ-42329001 (P)	*No significant effect*	9
$mGlu_2$	6x57	F780A (h)	JNJ-46281222 (P)	*No significant effect*	9
$mGlu_2$	6x57	F780A (h)	LY2607540 (P)	*No significant effect*	9
$mGlu_2$	6x57	F780A (h)	LY487379 (P)	*No significant effect*	9
$mGlu_2$	6x57	F780A (r)	RO4988546 (N)	*7.9, 20.8*	10
$mGlu_2$	6x57	F780A (r)	RO5488608 (N)	*0.8, 11.0*	10
$mGlu_2$	7x32	T793A (r)	RO4988546 (N)	*0.9*	10
$mGlu_2$	7x32	T793A (r)	RO5488608 (N)	*0.9*	10
$mGlu_2$	7x33	M794A (r)	RO4988546 (N)	*0.7, 2.9*	10
$mGlu_2$	7x33	M794A (r)	RO5488608 (N)	*1.1, 3.2*	10
$mGlu_2$	7x34	C795A (r)	RO4988546 (N)	*0.6*	10
$mGlu_2$	7x34	C795A (r)	RO5488608 (N)	*1.2*	10
$mGlu_2$	7x36	S797A (r)	RO4988546 (N)	*1.3*	10
$mGlu_2$	7x36	S797A (r)	RO5488608 (N)	*1.5*	10
$mGlu_2$	7x37	V798A (r)	RO4988546 (N)	*1.5, 5.7*	10
$mGlu_2$	7x37	V798A (r)	RO5488608 (N)	*5.5, 4.1*	10
$mGlu_4$	2x51	A638S (r)	MPEP (P)	*1.2*	14
$mGlu_4$	2x51	A638S (r)	PHCCC (P)	*1.2*	14
$mGlu_4$	2x51	A638S (r)	VU0155041 (P)	*0.34*	14

$mGlu_4$	2x51	A638S (r)	VU0415374	(P)	*1.8*	[14]
$mGlu_4$	2x52	T639I (r)	MPEP	(P)	*0.97*	[14]
$mGlu_4$	2x52	T639I (r)	PHCCC	(P)	*1.6*	[14]
$mGlu_4$	2x52	T639I (r)	VU0155041	(P)	*0.20*	[14]
$mGlu_4$	2x52	T639I (r)	VU0415374	(P)	*1.7*	[14]
$mGlu_4$	ECL1	E646A (r)	MPEP	(P)	*0.61*	[14]
$mGlu_4$	ECL1	E646A (r)	PHCCC	(P)	*0.69*	[14]
$mGlu_4$	ECL1	E646A (r)	VU0155041	(P)	*0.31*	[14]
$mGlu_4$	ECL1	E646A (r)	VU0415374	(P)	*1.3*	[14]
$mGlu_4$	3x31	L654F (r)	MPEP	(P)	*1.9*	[14]
$mGlu_4$	3x31	L654F (r)	PHCCC	(P)	*0.83*	[14]
$mGlu_4$	3x31	L654F (r)	VU0155041	(P)	*1.7*	[14]
$mGlu_4$	3x31	L654F (r)	VU0415374	(P)	*0.76*	[14]
$mGlu_4$	3x32	R655Q (r)	MPEP	(P)	*1.1*	[14]
$mGlu_4$	3x32	R655Q (r)	PHCCC	(P)	*1.1*	[14]
$mGlu_4$	3x32	R655Q (r)	VU0155041	(P)	*Abolished potentiation*	[14]
$mGlu_4$	3x32	R656A (r)	VU0155041	(P)	*21.0*	[14]
$mGlu_4$	3x32	R655Q (r)	VU0415374	(P)	*0.47*	[14]
$mGlu_4$	3x32	R656A (r)	VU0415374	(P)	*1.3*	[14]
$mGlu_4$	3x33	R656A (r)	MPEP	(P)	*1.7*	[14]
$mGlu_4$	3x33	R656A (r)	PHCCC	(P)	*0.61*	[14]
$mGlu_4$	3x40	M663P (r)	MPEP	(P)	*1.2*	[14]
$mGlu_4$	3x40	M663P (r)	PHCCC	(P)	*2.2*	[14]
$mGlu_4$	3x40	M663P (r)	VU0155041	(P)	*3.6*	[14]
$mGlu_4$	3x40	M663P (r)	VU0415374	(P)	*1.6*	[14]
$mGlu_4$	3x41	S664A (r)	MPEP	(P)	*1.1*	[14]
$mGlu_4$	3x41	S664C (r)	MPEP	(P)	*1.7*	[14]
$mGlu_4$	3x41	S664A (r)	PHCCC	(P)	*2.0*	[14]
$mGlu_4$	3x41	S664C (r)	PHCCC	(P)	*2.1*	[14]
$mGlu_4$	3x41	S664A (r)	VU0155041	(P)	*0.72*	[14]
$mGlu_4$	3x41	S664C (r)	VU0155041	(P)	*6.8*	[14]
$mGlu_4$	3x41	S664A (r)	VU0415374	(P)	*1.1*	[14]
$mGlu_4$	3x41	S664C (r)	VU0415374	(P)	*3.0*	[14]
$mGlu_4$	3x42	I665F (r)	MPEP	(P)	*3.7*	[14]
$mGlu_4$	3x42	I665F (r)	PHCCC	(P)	*2.4*	[14]
$mGlu_4$	3x42	I665F (r)	VU0155041	(P)	*0.49*	[14]
$mGlu_4$	3x42	I665F (r)	VU0415374	(P)	*1.7*	[14]
$mGlu_4$	3x44	Y667V (r)	MPEP	(P)	*2.2*	[14]
$mGlu_4$	3x44	Y667V (r)	PHCCC	(P)	*3.4*	[14]
$mGlu_4$	3x44	Y667V (r)	VU0155041	(P)	*1.2*	[14]
$mGlu_4$	3x44	Y667V (r)	VU0415374	(P)	*0.86*	[14]
$mGlu_4$	4x42	L709V (r)	MPEP	(P)	*1.8*	[14]
$mGlu_4$	4x42	L709V (r)	PHCCC	(P)	*2.0*	[14]

$mGlu_4$	4x42	L709V (r)	VU0155041	(P)	*1.3*	14
$mGlu_4$	4x42	L709V (r)	VU0415374	(P)	*2.0*	14
$mGlu_4$	4x45	L712G (r)	MPEP	(P)	*1.7*	14
$mGlu_4$	4x45	L712G (r)	PHCCC	(P)	*1.7*	14
$mGlu_4$	4x45	L712G (r)	VU0155041	(P)	*1.2*	14
$mGlu_4$	4x45	L712G (r)	VU0415374	(P)	*2.0*	14
$mGlu_4$	ECL2	S723P (r)	MPEP	(P)	*3.0*	14
$mGlu_4$	ECL2	S723P (r)	PHCCC	(P)	*2.0*	14
$mGlu_4$	ECL2	S723P (r)	VU0155041	(P)	*3.8*	14
$mGlu_4$	ECL2	S723P (r)	VU0415374	(P)	*3.2*	14
$mGlu_4$	5x39	S752G (r)	MPEP	(P)	*1.9*	14
$mGlu_4$	5x39	S752G (r)	PHCCC	(P)	*2.3*	14
$mGlu_4$	5x39	S752G (r)	VU0155041	(P)	*0.41*	14
$mGlu_4$	5x39	S752G (r)	VU0415374	(P)	*1.8*	14
$mGlu_4$	5x43	L756K (r)	MPEP	(P)	*0.83*	14
$mGlu_4$	5x43	L756S (r)	MPEP	(P)	*0.75*	14
$mGlu_4$	5x43	L756K (r)	PHCCC	(P)	*2.7*	14
$mGlu_4$	5x43	L756S (r)	PHCCC	(P)	*1.9*	14
$mGlu_4$	5x43	L756K (r)	VU0155041	(P)	*74.8*	14
$mGlu_4$	5x43	L756S (r)	VU0155041	(P)	*29.7*	14
$mGlu_4$	5x43	L756K (r)	VU0415374	(P)	*1.1*	14
$mGlu_4$	5x43	L756S (r)	VU0415374	(P)	*1.5*	14
$mGlu_4$	5x48	M761I (r)	MPEP	(P)	*1.4*	14
$mGlu_4$	5x48	M761I (r)	PHCCC	(P)	*1.4*	14
$mGlu_4$	5x48	M761I (r)	VU0155041	(P)	*2.6*	14
$mGlu_4$	5x48	M761I (r)	VU0415374	(P)	*1.0*	14
$mGlu_4$	6x49	V797I (r)	MPEP	(P)	*2.7*	14
$mGlu_4$	6x49	V797I (r)	PHCCC	(P)	*2.3*	14
$mGlu_4$	6x49	V797I (r)	VU0155041	(P)	*3.0*	14
$mGlu_4$	6x49	V797I (r)	VU0415374	(P)	*2.6*	14
$mGlu_4$	6x50	W798A (r)	MPEP	(P)	*2.2*	14
$mGlu_4$	6x50	W798A (r)	PHCCC	(P)	*Abolished potentiation*	14
$mGlu_4$	6x50	W798A (r)	VU0155041	(P)	*11.6*	14
$mGlu_4$	6x50	W798A (r)	VU0415374	(P)	*26.4*	14
$mGlu_4$	6x53	F801A (r)	MPEP	(P)	*8.2*	14
$mGlu_4$	6x53	F801A (r)	PHCCC	(P)	*0.65*	14
$mGlu_4$	6x53	F801A (r)	VU0155041	(P)	*7.0*	14
$mGlu_4$	6x53	F801A (r)	VU0415374	(P)	*19.2*	14
$mGlu_4$	7x37	V826M (r)	MPEP	(P)	*3.8*	14
$mGlu_4$	7x37	V826M (r)	PHCCC	(P)	*7.3*	14
$mGlu_4$	7x37	V826M (r)	VU0155041	(P)	*0.63*	14
$mGlu_4$	7x37	V826M (r)	VU0415374	(P)	*6.0*	14
$mGlu_4$	7x40	S829A (r)	MPEP	(P)	*6.0*	14

mGlu$_4$	7x40	S829A (r)	PHCCC (P)	*1.6*	14
mGlu$_4$	7x40	S829A (r)	VU0155041 (P)	*2.2*	14
mGlu$_4$	7x40	S829A (r)	VU0415374 (P)	*1.4*	14
mGlu$_4$	7x41	A830V (r)	MPEP (P)	*2.2*	14
mGlu$_4$	7x41	A830V (r)	PHCCC (P)	*2.3*	14
mGlu$_4$	7x41	A830V (r)	VU0155041 (P)	*0.59*	14
mGlu$_4$	7x41	A830V (r)	VU0415374 (P)	*1.2*	14
mGlu$_5$	1x41	I580V (r)	CPPHA (P)	*No significant effect*	1
mGlu$_5$	1x43	A582P (r)	CPPHA (P)	*No significant effect*	1
mGlu$_5$	1x46	F585I (r)	[^{3}H]methoxyPEPy (N)	**2.1**	15
mGlu$_5$	1x46	F585I (r)	CPPHA (P)	*Abolished potentiation*	1
mGlu$_5$	1x46	F585I (r)	CPPHA (P)	**3.0**	16
mGlu$_5$	1x46	F585I (r)	DPFE (P)	*No significant effect*	17
mGlu$_5$	1x46	F585I (r)	M-5MPEP (N)	*No significant effect*	17
mGlu$_5$	1x46	F585I (r)	MPEP (N)	**0.87**	15
mGlu$_5$	1x46	F585I (r)	MPEP (N)	**2.0**	16
mGlu$_5$	1x46	F585I (r)	VU0285683 (N)	*No significant effect*	17
mGlu$_5$	1x46	F585I (r)	VU0366058 (N)	*No significant effect*	17
mGlu$_5$	1x46	F585I (r)	VU0366248 (N)	*No significant effect*	17
mGlu$_5$	1x46	F585I (r)	VU0366249 (N)	*No significant effect*	17
mGlu$_5$	1x46	F585I (r)	VU0409106 (N)	*No significant effect*	17
mGlu$_5$	1x46	F585I (r)	VU29 (P)	*No significant effect*	1
mGlu$_5$	1x46	F585I (r)	VU29 (P)	*No significant effect*	17
mGlu$_5$	2x52	C630M (r)	CPPHA (P)	*No significant effect*	1
mGlu$_5$	2x53	T632A (h)	2-BisPEB (N)	*1.1*	18
mGlu$_5$	2x53	T632P (h)	2-BisPEB (N)	*0.16*	18
mGlu$_5$	2x53	T632A (h)	3-BisPEB (N)	*0.9*	18
mGlu$_5$	2x53	T632P (h)	3-BisPEB (N)	*0.35*	18
mGlu$_5$	2x53	T632A (h)	4-BisPEB (N)	*1.7*	18
mGlu$_5$	2x53	T632P (h)	4-BisPEB (N)	*1.2*	18
mGlu$_5$	2x53	T632A (h)	MPEP (N)	*0.56*	18
mGlu$_5$	2x53	T632P (h)	MPEP (N)	*0.25*	18
mGlu$_5$	2x56	L634F (r)	CPPHA (P)	*No significant effect*	1
mGlu$_5$	3x33	R647A (r)	[^{3}H]Fenobam (N)	**4.8**	19
mGlu$_5$	3x33	R647A (r)	[^{3}H]methoxyPEPy (N)	**1.0**	15
mGlu$_5$	3x33	R647A (r)	[^{3}H]MPEP (N)	**0.5**	20
mGlu$_5$	3x33	R648E (h)	2-BisPEB (N)	*0.15*	18
mGlu$_5$	3x33	R648E (h)	3-BisPEB (N)	*0.27*	18
mGlu$_5$	3x33	R648E (h)	4-BisPEB (N)	*1.0*	18
mGlu$_5$	3x33	R647A (r)	DFB (P)	*No significant effect*	21
mGlu$_5$	3x33	R647A (r)	DPFE (P)	*No significant effect*	17
mGlu$_5$	3x33	R647A (r)	Fenobam (N)	*1.9*	19
mGlu$_5$	3x33	R647A (r)	M-5MPEP (N)	*No significant effect*	17

$mGlu_5$	3x33	R647A (r)	MPEP	(N)	**0.49**	15
$mGlu_5$	3x33	R647A (r)	MPEP	(N)	*0.2*	20
$mGlu_5$	3x33	R648E (h)	MPEP	(N)	*0.11*	18
$mGlu_5$	3x33	R647A (r)	MTEP	(N)	*0.4*	19
$mGlu_5$	3x33	R647A (r)	VU0285683	(N)	*No significant effect*	17
$mGlu_5$	3x33	R647A (r)	VU0360172	(P)	**2.5**	15
$mGlu_5$	3x33	R647A (r)	VU0360173	(P)	**3.5**	15
$mGlu_5$	3x33	R647A (r)	VU0366058	(N)	*No significant effect*	17
$mGlu_5$	3x33	R647A (r)	VU0366248	(N)	*No significant effect*	17
$mGlu_5$	3x33	R647A (r)	VU0366249	(N)	*No significant effect*	17
$mGlu_5$	3x33	R647A (r)	VU0403602	(P)	**1.4**	15
$mGlu_5$	3x33	R647A (r)	VU0405386	(P)	**2.0**	15
$mGlu_5$	3x33	R647A (r)	VU0405398	(P)	**1.0**	15
$mGlu_5$	3x33	R647A (r)	VU0409106	(N)	*No significant effect*	17
$mGlu_5$	3x33	R647A (r)	VU0415051	(P)	**1.1**	15
$mGlu_5$	3x33	R647A (r)	VU29	(P)	*No significant effect*	17
$mGlu_5$	3x34	I649L (h)	[^{3}H]M-MPEP	(N)	**0.86**	8
$mGlu_5$	3x35	G650L (h)	[^{3}H]M-MPEP	(N)	**0.74**	8
$mGlu_5$	3x36	I651V (h)	[^{3}H]M-MPEP	(N)	**1.5**	8
$mGlu_5$	3x36	I650A (r)	[^{3}H]methoxyPEPy	(N)	**0.65**	15
$mGlu_5$	3x36	I651F (h)	2-BisPEB	(N)	*41*	18
$mGlu_5$	3x36	I651F (h)	3-BisPEB	(N)	*>20*	18
$mGlu_5$	3x36	I651F (h)	4-BisPEB	(N)	*>21*	18
$mGlu_5$	3x36	I650A (r)	DPFE	(P)	*No significant effect*	17
$mGlu_5$	3x36	I650A (r)	M-5MPEP	(N)	*No significant effect*	17
$mGlu_5$	3x36	I650A (r)	MPEP	(N)	**0.34, 0.91**	15
$mGlu_5$	3x36	I651F (h)	MPEP	(N)	*62*	18
$mGlu_5$	3x36	I650A (r)	VU0285683	(N)	*No significant effect*	17
$mGlu_5$	3x36	I650A (r)	VU0360172	(P)	**0.36**	15
$mGlu_5$	3x36	I650A (r)	VU0360173	(P)	**0.66**	15
$mGlu_5$	3x36	I650A (r)	VU0366058	(N)	*No significant effect*	17
$mGlu_5$	3x36	I650A (r)	VU0366248	(N)	*No significant effect*	17
$mGlu_5$	3x36	I650A (r)	VU0366249	(N)	*No significant effect*	17
$mGlu_5$	3x36	I650A (r)	VU0403602	(P)	**1.4, 2.3**	15
$mGlu_5$	3x36	I650A (r)	VU0405386	(P)	**0.87, 1.9**	15
$mGlu_5$	3x36	I650A (r)	VU0405398	(P)	**0.45, 0.44**	15
$mGlu_5$	3x36	I650A (r)	VU0409106	(N)	*No significant effect*	17
$mGlu_5$	3x36	I650A (r)	VU0415051	(P)	**1.8**	15
$mGlu_5$	3x36	I650A (r)	VU29	(P)	*No significant effect*	17
$mGlu_5$	3x37	G651F (r)	[^{3}H]methoxyPEPy	(N)	**Abolished binding**	15
$mGlu_5$	3x37	G651F (r)	MPEP	(N)	**11.2**	15
$mGlu_5$	3x37	G651F (r)	VU0360172	(P)	**6.6**	15
$mGlu_5$	3x37	G651F (r)	VU0360173	(P)	**Abolished potentiation**	15

mGlu$_5$	3x37	G651F (r)	VU0403602 (P)	**42.7**	15
mGlu$_5$	3x37	G651F (r)	VU0405386 (P)	**7.9**	15
mGlu$_5$	3x37	G651F (r)	VU0405398 (P)	**8.9**	15
mGlu$_5$	3x37	G651F (r)	VU0415051 (P)	**2.5**	15
mGlu$_5$	3x38	L652T (r)	CPPHA (P)	*No significant effect*	1
mGlu$_5$	3x39	S653A (r)	CPPHA (P)	*No significant effect*	1
mGlu$_5$	3x39	S653A (r)	DPFE (P)	*No significant effect*	17
mGlu$_5$	3x39	S653A (r)	M-5MPEP (N)	*No significant effect*	17
mGlu$_5$	3x39	S653A (r)	MPEP (N)	*No significant effect*	15
mGlu$_5$	3x39	S653A (r)	VU0285683 (N)	*No significant effect*	17
mGlu$_5$	3x39	S653A (r)	VU0366058 (N)	*No significant effect*	17
mGlu$_5$	3x39	S653A (r)	VU0366248 (N)	*No significant effect*	17
mGlu$_5$	3x39	S653A (r)	VU0366249 (N)	*No significant effect*	17
mGlu$_5$	3x39	S653A (r)	VU0409106 (N)	*No significant effect*	17
mGlu$_5$	3x39	S653A (r)	VU29 (P)	*No significant effect*	17
mGlu$_5$	3x40	P654S (r)	[^{3}H]Fenobam (N)	**Abolished binding**	19
mGlu$_5$	3x40	P655S (h)	[^{3}H]M-MPEP (N)	**7.7**	8
mGlu$_5$	3x40	P654F (r)	[^{3}H]methoxyPEPy (N)	**Abolished binding**	15
mGlu$_5$	3x40	P654S (r)	[^{3}H]methoxyPEPy (N)	**Abolished binding**	15
mGlu$_5$	3x40	P654S (r)	[^{3}H]MPEP (N)	**40.0**	20
mGlu$_5$	3x40	P655S (h)	2-BisPEB (N)	*2.0*	18
mGlu$_5$	3x40	P655S (h)	3-BisPEB (N)	*>20*	18
mGlu$_5$	3x40	P655S (h)	4-BisPEB (N)	*1.5*	18
mGlu$_5$	3x40	P654S (r)	DFB (P)	*No significant effect*	21
mGlu$_5$	3x40	P654F (r)	DPFE (P)	**1.2**	17
mGlu$_5$	3x40	P654S (r)	DPFE (P)	**9.1**	17
mGlu$_5$	3x40	P654S (r)	Fenobam (N)	*7.9*	19
mGlu$_5$	3x40	P654F (r)	M-5MPEP (N)	**>338.8**	17
mGlu$_5$	3x40	P654S (r)	M-5MPEP (N)	**30.2**	17
mGlu$_5$	3x40	P654F (r)	MPEP (N)	**29512**	15
mGlu$_5$	3x40	P654S (r)	MPEP (N)	**30.2**	15
mGlu$_5$	3x40	P654S (r)	MPEP (N)	*15*	20
mGlu$_5$	3x40	P655S (h)	MPEP (N)	*7.4,*	18
mGlu$_5$	3x40	P654S (r)	MTEP (N)	*>149*	19
mGlu$_5$	3x40	P654F (r)	VU0285683 (N)	**Abolished inhibition**	17
mGlu$_5$	3x40	P654S (r)	VU0285683 (N)	**8.1**	17
mGlu$_5$	3x40	P654F (r)	VU0360172 (P)	**5.9**	15
mGlu$_5$	3x40	P654S (r)	VU0360172 (P)	**1.9**	15
mGlu$_5$	3x40	P654F (r)	VU0360173 (P)	**Abolished potentiation**	15
mGlu$_5$	3x40	P654S (r)	VU0360173 (P)	**>2.8**	15
mGlu$_5$	3x40	P654F (r)	VU0366058 (N)	**Abolished inhibition**	17
mGlu$_5$	3x40	P654S (r)	VU0366058 (N)	**2.0**	17
mGlu$_5$	3x40	P654F (r)	VU0366248 (N)	**Abolished inhibition**	17

$mGlu_5$	3x40	P654S (r)	VU0366248	(N)	**14.1**	17
$mGlu_5$	3x40	P654F (r)	VU0366249	(N)	**Abolished inhibition**	17
$mGlu_5$	3x40	P654S (r)	VU0366249	(N)	**>27.5**	17
$mGlu_5$	3x40	P654F (r)	VU0403602	(P)	**138.0**	15
$mGlu_5$	3x40	P654S (r)	VU0403602	(P)	**6.3**	15
$mGlu_5$	3x40	P654F (r)	VU0405386	(P)	**64.6**	15
$mGlu_5$	3x40	P654S (r)	VU0405386	(P)	**1.7**	15
$mGlu_5$	3x40	P654F (r)	VU0405398	(P)	**40.7**	15
$mGlu_5$	3x40	P654S (r)	VU0405398	(P)	**13.8**	15
$mGlu_5$	3x40	P654F (r)	VU0409106	(N)	**Abolished inhibition**	17
$mGlu_5$	3x40	P654S (r)	VU0409106	(N)	**1.5**	17
$mGlu_5$	3x40	P654F (r)	VU0415051	(P)	**123.0**	15
$mGlu_5$	3x40	P654S (r)	VU0415051	(P)	**44.7**	15
$mGlu_5$	3x40	P654F (r)	VU29	(P)	**Abolished potentiation**	17
$mGlu_5$	3x40	P654S (r)	VU29	(P)	**2.6**	17
$mGlu_5$	3x43	S657C (r)	[^{3}H]Fenobam	(N)	**Abolished binding**	19
$mGlu_5$	3x43	S658C (h)	[^{3}H]M-MPEP	(N)	**4.7**	8
$mGlu_5$	3x43	S657C (r)	[^{3}H]methoxyPEPy	(N)	**0.78**	15
$mGlu_5$	3x43	S657C (r)	[^{3}H]MPEP	(N)	**0.9**	20
$mGlu_5$	3x43	S657C (r)	DFB	(P)	*Abolished potentiation*	21
$mGlu_5$	3x43	S657A (r)	DPFE	(P)	*No significant effect*	17
$mGlu_5$	3x43	S657C (r)	DPFE	(P)	*No significant effect*	17
$mGlu_5$	3x43	S657C (r)	Fenobam	(N)	*>178*	19
$mGlu_5$	3x43	S657A (r)	M-5MPEP	(N)	**0.28**	17
$mGlu_5$	3x43	S657C (r)	M-5MPEP	(N)	**2.4**	17
$mGlu_5$	3x43	S657A (r)	MPEP	(N)	**0.60**	17
$mGlu_5$	3x43	S657C (r)	MPEP	(N)	**0.56, 1.8**	15
$mGlu_5$	3x43	S657C (r)	MPEP	(N)	*1.4*	20
$mGlu_5$	3x43	S657C (r)	MTEP	(N)	*1.6*	19
$mGlu_5$	3x43	S657A (r)	VU0285683	(N)	*No significant effect*	17
$mGlu_5$	3x43	S657C (r)	VU0285683	(N)	*No significant effect*	17
$mGlu_5$	3x43	S657A (r)	VU0366058	(N)	*No significant effect*	17
$mGlu_5$	3x43	S657C (r)	VU0366058	(N)	*No significant effect*	17
$mGlu_5$	3x43	S657A (r)	VU0366248	(N)	**0.35**	17
$mGlu_5$	3x43	S657C (r)	VU0366248	(N)	**16.2**	17
$mGlu_5$	3x43	S657A (r)	VU0366249	(N)	**0.25**	17
$mGlu_5$	3x43	S657C (r)	VU0366249	(N)	**1.2**	17
$mGlu_5$	3x43	S657C (r)	VU0403602	(P)	**5.2**	15
$mGlu_5$	3x43	S657C (r)	VU0405386	(P)	**2.3**	15
$mGlu_5$	3x43	S657C (r)	VU0405398	(P)	**0.89**	15
$mGlu_5$	3x43	S657A (r)	VU0409106	(N)	*No significant effect*	17
$mGlu_5$	3x43	S657C (r)	VU0409106	(N)	*No significant effect*	17
$mGlu_5$	3x43	S657C (r)	VU0415051	(P)	**3.6**	15

mGlu$_5$	3x43	S657A (r)	VU29 (P)	*No significant effect*	17
mGlu$_5$	3x43	S657C (r)	VU29 (P)	*No significant effect*	17
mGlu$_5$	3x44	Y658F (r)	[^{3}H]Fenobam (N)	**0.2**	19
mGlu$_5$	3x44	Y658V (r)	[^{3}H]Fenobam (N)	**Abolished binding**	19
mGlu$_5$	3x44	Y658V (r)	[^{3}H]methoxyPEPy (N)	**Abolished binding**	15
mGlu$_5$	3x44	Y658F (r)	[^{3}H]MPEP (N)	**0.8**	20
mGlu$_5$	3x44	Y658V (r)	[^{3}H]MPEP (N)	**Abolished binding**	20
mGlu$_5$	3x44	Y659A (h)	2-BisPEB (N)	*14*	18
mGlu$_5$	3x44	Y659F (h)	2-BisPEB (N)	*0.48*	18
mGlu$_5$	3x44	Y659A (h)	3-BisPEB (N)	*>20*	18
mGlu$_5$	3x44	Y659F (h)	3-BisPEB (N)	*0.54*	18
mGlu$_5$	3x44	Y659A (h)	4-BisPEB (N)	*>21*	18
mGlu$_5$	3x44	Y659F (h)	4-BisPEB (N)	*0.30*	18
mGlu$_5$	3x44	Y658F (r)	DFB (P)	*No significant effect*	21
mGlu$_5$	3x44	Y658V (r)	DFB (P)	*No significant effect*	21
mGlu$_5$	3x44	Y658V (r)	DPFE (P)	**6.0**	17
mGlu$_5$	3x44	Y658F (r)	Fenobam (N)	*0.2*	19
mGlu$_5$	3x44	Y658V (r)	Fenobam (N)	*18.0*	19
mGlu$_5$	3x44	Y658V (r)	M-5MPEP (N)	**>338.8**	17
mGlu$_5$	3x44	Y658F (r)	MPEP (N)	*0.7*	20
mGlu$_5$	3x44	Y658V (r)	MPEP (N)	*Decreased inhibition*	15
mGlu$_5$	3x44	Y658V (r)	MPEP (N)	**102.3**	16
mGlu$_5$	3x44	Y658V (r)	MPEP (N)	*54.0*	20
mGlu$_5$	3x44	Y659A (h)	MPEP (N)	*120*	18
mGlu$_5$	3x44	Y659F (h)	MPEP (N)	*0.68*	18
mGlu$_5$	3x44	Y658V (r)	VU0285683 (N)	**16.2**	17
mGlu$_5$	3x44	Y658V (r)	VU0360172 (P)	**Abolished potentiation**	15
mGlu$_5$	3x44	Y658V (r)	VU0360173 (P)	**Abolished potentiation**	15
mGlu$_5$	3x44	Y658V (r)	VU0366058 (N)	**38.9**	17
mGlu$_5$	3x44	Y658V (r)	VU0366248 (N)	**34.7**	17
mGlu$_5$	3x44	Y658V (r)	VU0366249 (N)	**Abolished inhibition**	17
mGlu$_5$	3x44	Y658V (r)	VU0403602 (P)	**Abolished potentiation**	15
mGlu$_5$	3x44	Y658V (r)	VU0405386 (P)	**Abolished potentiation**	15
mGlu$_5$	3x44	Y658V (r)	VU0405398 (P)	**93.3**, *PAM to NAM switch*	15
mGlu$_5$	3x44	Y658V (r)	VU0409106 (N)	**25.7**	17
mGlu$_5$	3x44	Y658V (r)	VU0415051 (P)	**Abolished potentiation**	15
mGlu$_5$	3x44	Y658V (r)	VU0424465 (P)	**53.7**	22
mGlu$_5$	3x44	Y658V (r)	VU0430644 (P)	*PAM to NAM switch*	22
mGlu$_5$	3x44	Y658V (r)	VU0430644 (P)	**77.6**	22
mGlu$_5$	3x44	Y658V (r)	VU0465731 (P)	**0.47**	22
mGlu$_5$	3x44	Y658V (r)	VU29 (P)	**0.74**	17
mGlu$_5$	4x48	V708A (r)	CPPHA (P)	*No significant effect*	1
mGlu$_5$	4x50	L710W (r)	CPPHA (P)	*No significant effect*	1

mGlu$_5$	4x52	I712V (r)	CPPHA (P)	*No significant effect*	1
mGlu$_5$	4x53	M713V (r)	CPPHA (P)	*No significant effect*	1
mGlu$_5$	45x51	N733A (r)	[^{3}H]Fenobam (N)	**1.0**	19
mGlu$_5$	45x51	N733A (r)	[^{3}H]MPEP (N)	**1.1**	20
mGlu$_5$	45x51	N733A (r)	DFB (P)	*Potency increase abolished - efficacy increase remained*	21
mGlu$_5$	45x51	N733A (r)	Fenobam (N)	*0.9*	19
mGlu$_5$	45x51	N733A (r)	MPEP (N)	*1.2*	20
mGlu$_5$	5x38	L737A (r)	CPPHA (P)	*No significant effect*	1
mGlu$_5$	5x39	G738S (r)	CPPHA (P)	*No significant effect*	1
mGlu$_5$	5x40	V739M (r)	[^{3}H]methoxyPEPy (N)	**1.1**	15
mGlu$_5$	5x40	V739M (r)	CPPHA (P)	*No significant effect*	1
mGlu$_5$	5x40	V739M (r)	DPFE (P)	*No significant effect*	17
mGlu$_5$	5x40	V739M (r)	M-5MPEP (N)	*No significant effect*	17
mGlu$_5$	5x40	V739M (r)	MPEP (N)	**0.91**	15
mGlu$_5$	5x40	V739M (r)	VU0285683 (N)	*No significant effect*	17
mGlu$_5$	5x40	V739M (r)	VU0366058 (N)	*No significant effect*	17
mGlu$_5$	5x40	V739M (r)	VU0366248 (N)	*No significant effect*	17
mGlu$_5$	5x40	V739M (r)	VU0366249 (N)	*No significant effect*	17
mGlu$_5$	5x40	V739M (r)	VU0409106 (N)	*No significant effect*	17
mGlu$_5$	5x40	V739M (r)	VU29 (P)	*No significant effect*	17
mGlu$_5$	5x41	V740L (r)	CPPHA (P)	*No significant effect*	1
mGlu$_5$	5x43	P742S (r)	[^{3}H]methoxyPEPy (N)	**3.4**	15
mGlu$_5$	5x43	P742S (r)	DPFE (P)	**26.0**	17
mGlu$_5$	5x43	P742S (r)	M-5MPEP (N)	**3.0**	17
mGlu$_5$	5x43	P742S (r)	MPEP (N)	**3.2, 1.6**	15
mGlu$_5$	5x43	P742S (r)	VU0285683 (N)	**1.1**	17
mGlu$_5$	5x43	P742S (r)	VU0360172 (P)	**1.3, 4.7**	15
mGlu$_5$	5x43	P742S (r)	VU0360173 (P)	**6.3, 1.0**	15
mGlu$_5$	5x43	P742S (r)	VU0366058 (N)	*No significant effect*	17
mGlu$_5$	5x43	P742S (r)	VU0366248 (N)	*No significant effect*	17
mGlu$_5$	5x43	P742S (r)	VU0366249 (N)	**Abolished inhibition**	17
mGlu$_5$	5x43	P742S (r)	VU0403602 (P)	**4.3, 10.5**	15
mGlu$_5$	5x43	P742S (r)	VU0405386 (P)	**1.2, 6.2**	15
mGlu$_5$	5x43	P742S (r)	VU0405398 (P)	**1.3, 1.9**	15
mGlu$_5$	5x43	P742S (r)	VU0409106 (N)	*No significant effect*	17
mGlu$_5$	5x43	P742S (r)	VU0415051 (P)	**1.8, 3.4**	15
mGlu$_5$	5x43	P742S (r)	VU29 (P)	**2.5**	17
mGlu$_5$	5x44	L743A (r)	[^{3}H]Fenobam (N)	**0.6**	19
mGlu$_5$	5x44	L743V (r)	[^{3}H]Fenobam (N)	**1.1**	19
mGlu$_5$	5x44	L743V (r)	[^{3}H]methoxyPEPy (N)	**2.1**	15
mGlu$_5$	5x44	L743A (r)	[^{3}H]MPEP (N)	**4.7**	20
mGlu$_5$	5x44	L743V (r)	[^{3}H]MPEP (N)	**3.9**	20

mGlu$_5$	5x44	L743A (r)	DFB (P)	*Increased potentiation*	21
mGlu$_5$	5x44	L743V (r)	DFB (P)	*Increased potentiation*	21
mGlu$_5$	5x44	L743V (r)	DPFE (P)	**0.06**	17
mGlu$_5$	5x44	L743A (r)	Fenobam (N)	*0.7*	19
mGlu$_5$	5x44	L743V (r)	Fenobam (N)	*0.6*	19
mGlu$_5$	5x44	L743V (r)	M-5MPEP (N)	*No significant effect*	17
mGlu$_5$	5x44	L743A (r)	MPEP (N)	*5.3*	20
mGlu$_5$	5x44	L743V (r)	MPEP (N)	**4.4**	15
mGlu$_5$	5x44	L743V (r)	MPEP (N)	**3.5**	16
mGlu$_5$	5x44	L743V (r)	MPEP (N)	*3.2*	20
mGlu$_5$	5x44	L743V (r)	Ro 67-7476 (P)	*Weak gain of potentiation (does not potentiate WT rat mGlu5)*	5
mGlu$_5$	5x44	L743V (r)	VU0285683 (N)	*No significant effect*	17
mGlu$_5$	5x44	L743V (r)	VU0360172 (P)	**0.51, 1.8**	15
mGlu$_5$	5x44	L743V (r)	VU0360173 (P)	**0.98, 1.8**	15
mGlu$_5$	5x44	L743V (r)	VU0366058 (N)	*No significant effect*	17
mGlu$_5$	5x44	L743V (r)	VU0366248 (N)	*No significant effect*	17
mGlu$_5$	5x44	L743V (r)	VU0366249 (N)	*No significant effect*	17
mGlu$_5$	5x44	L743V (r)	VU0403602 (P)	**1.6, 19.1**	15
mGlu$_5$	5x44	L743V (r)	VU0405386 (P)	**0.48, 2.5**	15
mGlu$_5$	5x44	L743V (r)	VU0405398 (P)	**1.5, 2.2**	15
mGlu$_5$	5x44	L743V (r)	VU0409106 (N)	*No significant effect*	17
mGlu$_5$	5x44	L743V (r)	VU0415051 (P)	**0.39, 4.3**	15
mGlu$_5$	5x44	L743V (r)	VU29 (P)	**2.2**	16
mGlu$_5$	5x44	L743V (r)	VU29 (P)	**2.3**	17
mGlu$_5$	5x47	N746A (r)	[^{3}H]methoxyPEPy (N)	**3.3**	15
mGlu$_5$	5x47	N747A (h)	2-BisPEB (N)	*11*	18
mGlu$_5$	5x47	N747S (h)	2-BisPEB (N)	*5.4*	18
mGlu$_5$	5x47	N747A (h)	3-BisPEB (N)	*>20*	18
mGlu$_5$	5x47	N747S (h)	3-BisPEB (N)	*11*	18
mGlu$_5$	5x47	N747A (h)	4-BisPEB (N)	*1.3*	18
mGlu$_5$	5x47	N747S (h)	4-BisPEB (N)	*2.4*	18
mGlu$_5$	5x47	N746A (r)	DPFE (P)	*No significant effect*	17
mGlu$_5$	5x47	N746A (r)	M-5MPEP (N)	**3.0**	17
mGlu$_5$	5x47	N746A (r)	MPEP (N)	**1.9, 1.9**	15
mGlu$_5$	5x47	N747A (h)	MPEP (N)	*1.6*	18
mGlu$_5$	5x47	N747S (h)	MPEP (N)	*4.2*	18
mGlu$_5$	5x47	N746A (r)	VU0285683 (N)	**5.6**	17
mGlu$_5$	5x47	N746A (r)	VU0360172 (P)	**1.0, 2.2**	15
mGlu$_5$	5x47	N746A (r)	VU0360173 (P)	**3.1**	15
mGlu$_5$	5x47	N746A (r)	VU0366058 (N)	**1.0**	17
mGlu$_5$	5x47	N746A (r)	VU0366248 (N)	**19.5**	17
mGlu$_5$	5x47	N746A (r)	VU0366249 (N)	**>27.5**	17

mGlu$_5$	5x47	N746A (r)	VU0403602	(P)	**1.4, 5.9**	[15]
mGlu$_5$	5x47	N746A (r)	VU0405386	(P)	**0.95, 3.4**	[15]
mGlu$_5$	5x47	N746A (r)	VU0405398	(P)	**2.8, 2.0**	[15]
mGlu$_5$	5x47	N746A (r)	VU0409106	(N)	**15.1**	[17]
mGlu$_5$	5x47	N746A (r)	VU0415051	(P)	**3.4**	[15]
mGlu$_5$	5x47	N746A (r)	VU29	(P)	*No significant effect*	[17]
mGlu$_5$	5x48	G747V (r)	[^{3}H]methoxyPEPy	(N)	**0.74**	[15]
mGlu$_5$	5x48	G748L (h)	2-BisPEB	(N)	*1.0*	[18]
mGlu$_5$	5x48	G748L (h)	3-BisPEB	(N)	*1.8*	[18]
mGlu$_5$	5x48	G748L (h)	4-BisPEB	(N)	*1.9*	[18]
mGlu$_5$	5x48	G747V (r)	DPFE	(P)	**0.56**	[17]
mGlu$_5$	5x48	G747V (r)	M-5MPEP	(N)	**1.0**	[17]
mGlu$_5$	5x48	G747V (r)	MPEP	(N)	**0.98**	[15]
mGlu$_5$	5x48	G748L (h)	MPEP	(N)	*4.9*	[18]
mGlu$_5$	5x48	G747V (r)	VU0285683	(N)	**0.74**	[17]
mGlu$_5$	5x48	G747V (r)	VU0360172	(P)	**0.65**	[15]
mGlu$_5$	5x48	G747V (r)	VU0366058	(N)	**0.17**	[17]
mGlu$_5$	5x48	G747V (r)	VU0366248	(N)	**0.41**	[17]
mGlu$_5$	5x48	G747V (r)	VU0366249	(N)	**0.26**	[17]
mGlu$_5$	5x48	G747V (r)	VU0405386	(P)	**0.65**	[15]
mGlu$_5$	5x48	G747V (r)	VU0409106	(N)	**0.95**	[17]
mGlu$_5$	5x48	G747V (r)	VU0415051	(P)	**0.66**	[15]
mGlu$_5$	5x48	G747V (r)	VU29	(P)	**29.5**	[17]
mGlu$_5$	6x45	T779A (r)	[^{3}H]methoxyPEPy	(N)	**1.2**	[15]
mGlu$_5$	6x45	T779A (r)	DPFE	(P)	*No significant effect*	[17]
mGlu$_5$	6x45	T779A (r)	M-5MPEP	(N)	*No significant effect*	[17]
mGlu$_5$	6x45	T779A (r)	MPEP	(N)	*Decreased inhibition*	[15]
mGlu$_5$	6x45	T779A (r)	VU0285683	(N)	*No significant effect*	[17]
mGlu$_5$	6x45	T779A (r)	VU0366058	(N)	*No significant effect*	[17]
mGlu$_5$	6x45	T779A (r)	VU0366248	(N)	*No significant effect*	[17]
mGlu$_5$	6x45	T779A (r)	VU0366249	(N)	*No significant effect*	[17]
mGlu$_5$	6x45	T779A (r)	VU0409106	(N)	*No significant effect*	[17]
mGlu$_5$	6x45	T779A (r)	VU29	(P)	*No significant effect*	[17]
mGlu$_5$	6x46	T780A (r)	[^{3}H]Fenobam	(N)	**Abolished binding**	[19]
mGlu$_5$	6x46	T780A (r)	[^{3}H]methoxyPEPy	(N)	**Abolished binding**	[15]
mGlu$_5$	6x46	T780A (r)	[^{3}H]MPEP	(N)	**4.6**	[20]
mGlu$_5$	6x46	T780A (r)	DFB	(P)	*Abolished potentiation*	[21]
mGlu$_5$	6x46	T780A (r)	DPFE	(P)	**70.8**	[17]
mGlu$_5$	6x46	T780A (r)	Fenobam	(N)	*>54*	[19]
mGlu$_5$	6x46	T780A (r)	M-5MPEP	(N)	**3.02**	[17]
mGlu$_5$	6x46	T780A (r)	MPEP	(N)	**16.6**	[15]
mGlu$_5$	6x46	T780A (r)	MPEP	(N)	*9.2*	[20]
mGlu$_5$	6x46	T780A (r)	VU0285683	(N)	**10.5**	[17]

$mGlu_5$	6x46	T780A (r)	VU0360172	(P)	**Abolished potentiation**	15
$mGlu_5$	6x46	T780A (r)	VU0360173	(P)	**Abolished potentiation**	15
$mGlu_5$	6x46	T780A (r)	VU0366058	(N)	**0.93**	17
$mGlu_5$	6x46	T780A (r)	VU0366248	(N)	**3.7**	17
$mGlu_5$	6x46	T780A (r)	VU0366249	(N)	**>27.5**	17
$mGlu_5$	6x46	T780A (r)	VU0403602	(P)	**125.9**	15
$mGlu_5$	6x46	T780A (r)	VU0405386	(P)	**524.8**	15
$mGlu_5$	6x46	T780A (r)	VU0405398	(P)	**22.4**	15
$mGlu_5$	6x46	T780A (r)	VU0409106	(N)	**8.1**	17
$mGlu_5$	6x46	T780A (r)	VU0415051	(P)	**33.1**, *PAM to NAM switch*	15
$mGlu_5$	6x46	T780A (r)	VU0424465	(P)	**10.5**	22
$mGlu_5$	6x46	T780A (r)	VU0430644	(P)	**134.9**	22
$mGlu_5$	6x46	T780A (r)	VU0465731	(P)	**31.6**	22
$mGlu_5$	6x46	T780A (r)	VU29	(P)	**7.8**	17
$mGlu_5$	6x49	I783A (r)	DPFE	(P)	*No significant effect*	17
$mGlu_5$	6x49	I783A (r)	M-5MPEP	(N)	*No significant effect*	17
$mGlu_5$	6x49	I783A (r)	MPEP	(N)	*No significant effect*	15
$mGlu_5$	6x49	I783A (r)	VU0285683	(N)	*No significant effect*	17
$mGlu_5$	6x49	I783A (r)	VU0366058	(N)	*No significant effect*	17
$mGlu_5$	6x49	I783A (r)	VU0366248	(N)	*No significant effect*	17
$mGlu_5$	6x49	I783A (r)	VU0366249	(N)	*No significant effect*	17
$mGlu_5$	6x49	I783A (r)	VU0409106	(N)	*No significant effect*	17
$mGlu_5$	6x49	I783A (r)	VU29	(P)	*No significant effect*	17
$mGlu_5$	6x50	W784A (r)	[^{3}H]Fenobam	(N)	**Abolished binding**	19
$mGlu_5$	6x50	W784F (r)	[^{3}H]Fenobam	(N)	**2.9**	19
$mGlu_5$	6x50	W784A (r)	[^{3}H]methoxyPEPy	(N)	**Abolished binding**	15
$mGlu_5$	6x50	W784A (r)	[^{3}H]MPEP	(N)	**Abolished binding**	20
$mGlu_5$	6x50	W784F (r)	[^{3}H]MPEP	(N)	**3.3**	20
$mGlu_5$	6x50	W785A (h)	2-BisPEB	(N)	*11*	18
$mGlu_5$	6x50	W785A (h)	3-BisPEB	(N)	*2.0*	18
$mGlu_5$	6x50	W785A (h)	4-BisPEB	(N)	*2.9*	18
$mGlu_5$	6x50	W784A (r)	DFB	(P)	*Increased potentiation*	21
$mGlu_5$	6x50	W784F (r)	DFB	(P)	*Increased potentiation*	21
$mGlu_5$	6x50	W784A (r)	DPFE	(P)	**15.5**	17
$mGlu_5$	6x50	W784A (r)	Fenobam	(N)	*>179*	19
$mGlu_5$	6x50	W784F (r)	Fenobam	(N)	*2.7*	19
$mGlu_5$	6x50	W784A (r)	M-5MPEP	(N)	**Abolished inhibition**	17
$mGlu_5$	6x50	W784A (r)	MPEP	(N)	**1202**	15
$mGlu_5$	6x50	W784A (r)	MPEP	(N)	*21.0*	20
$mGlu_5$	6x50	W784F (r)	MPEP	(N)	*2.0*	20
$mGlu_5$	6x50	W785A (h)	MPEP	(N)	*280*	18
$mGlu_5$	6x50	W784A (r)	VU0285683	(N)	**229.1**	17
$mGlu_5$	6x50	W784A (r)	VU0360172	(P)	**0.83**	15

mGlu$_5$	6x50	W784A (r)	VU0360173 (P)	**2.6**	15
mGlu$_5$	6x50	W784A (r)	VU0366058 (N)	**12.3**	17
mGlu$_5$	6x50	W784A (r)	VU0366248 (N)	**12.9**	17
mGlu$_5$	6x50	W784A (r)	VU0366249 (N)	**1.0**	17
mGlu$_5$	6x50	W784A (r)	VU0403602 (P)	**11.0**	15
mGlu$_5$	6x50	W784A (r)	VU0405386 (P)	**3.2**	15
mGlu$_5$	6x50	W784A (r)	VU0405398 (P)	**5.1**	15
mGlu$_5$	6x50	W784A (r)	VU0409106 (N)	**6.6**	17
mGlu$_5$	6x50	W784A (r)	VU0415051 (P)	**1.6**	15
mGlu$_5$	6x50	W784A (r)	VU0424465 (P)	**1.1**	22
mGlu$_5$	6x50	W784A (r)	VU0430644 (P)	**1.7**	22
mGlu$_5$	6x50	W784A (r)	VU0465731 (P)	*Gain in allosteric agonist activity*	22
mGlu$_5$	6x50	W784A (r)	VU0465731 (P)	**7.6**	22
mGlu$_5$	6x50	W784A (r)	VU29 (P)	**0.13**	17
mGlu$_5$	6x53	F787A (r)	[^{3}H]Fenobam (N)	**Abolished binding**	19
mGlu$_5$	6x53	F787A (r)	[^{3}H]MPEP (N)	**Abolished binding**	20
mGlu$_5$	6x53	F788A (h)	2-BisPEB (N)	*1.2*	18
mGlu$_5$	6x53	F788W (h)	2-BisPEB (N)	*3.3*	18
mGlu$_5$	6x53	F788A (h)	3-BisPEB (N)	*0.86*	18
mGlu$_5$	6x53	F788W (h)	3-BisPEB (N)	*>20*	18
mGlu$_5$	6x53	F788A (h)	4-BisPEB (N)	*≈21*	18
mGlu$_5$	6x53	F788W (h)	4-BisPEB (N)	*>21*	18
mGlu$_5$	6x53	F787A (r)	DFB (P)	*PAM to NAM switch*	21
mGlu$_5$	6x53	F787A (r)	DPFE (P)	**1.2**	17
mGlu$_5$	6x53	F787A (r)	Fenobam (N)	*42.0*	19
mGlu$_5$	6x53	F787A (r)	M-5MPEP (N)	**2.0**	17
mGlu$_5$	6x53	F787A (r)	MPEP (N)	*10.0*	20
mGlu$_5$	6x53	F788A (h)	MPEP (N)	*27*	18
mGlu$_5$	6x53	F788W (h)	MPEP (N)	*9.8*	18
mGlu$_5$	6x53	F787A (r)	VU0285683 (N)	**97.7**	17
mGlu$_5$	6x53	F787A (r)	VU0366058 (N)	**0.07**	17
mGlu$_5$	6x53	F787A (r)	VU0366248 (N)	**Abolished inhibition**	17
mGlu$_5$	6x53	F787A (r)	VU0366249 (N)	**Abolished inhibition**	17
mGlu$_5$	6x53	F787A (r)	VU0409106 (N)	**21.9**	17
mGlu$_5$	6x53	F787A (r)	VU29 (P)	**2.0**	17
mGlu$_5$	6x54	V788M (r)	[^{3}H]Fenobam (N)	**1.2**	19
mGlu$_5$	6x54	V788A (r)	[^{3}H]methoxyPEPy (N)	**1.3**	15
mGlu$_5$	6x54	V788M (r)	[^{3}H]MPEP (N)	**1.1**	20
mGlu$_5$	6x54	V788M (r)	DFB (P)	*No significant effect*	21
mGlu$_5$	6x54	V788A (r)	DPFE (P)	**0.07**	17
mGlu$_5$	6x54	V788M (r)	Fenobam (N)	*1.2*	19
mGlu$_5$	6x54	V788A (r)	M-5MPEP (N)	**0.93**	17
mGlu$_5$	6x54	F787A (r)	MPEP (N)	**60.3**	17

$mGlu_5$	6x54	V788A (r)	MPEP	(N)	**4.9, 1.4**	15
$mGlu_5$	6x54	V788M (r)	MPEP	(N)	*1.7*	20
$mGlu_5$	6x54	V788A (r)	VU0285683	(N)	**0.63**	17
$mGlu_5$	6x54	V788A (r)	VU0360172	(P)	**0.25, 0.31**	15
$mGlu_5$	6x54	V788A (r)	VU0360173	(P)	**0.15, 0.10**	15
$mGlu_5$	6x54	V788A (r)	VU0366058	(N)	**0.18**	17
$mGlu_5$	6x54	V788A (r)	VU0366248	(N)	**2.0**	17
$mGlu_5$	6x54	V788A (r)	VU0366249	(N)	**0.13**	17
$mGlu_5$	6x54	V788A (r)	VU0403602	(P)	**0.50, 0.36**	15
$mGlu_5$	6x54	V788A (r)	VU0405386	(P)	**0.18, 0.06**	15
$mGlu_5$	6x54	V788A (r)	VU0405398	(P)	**0.13, 0.02**	15
$mGlu_5$	6x54	V788A (r)	VU0409106	(N)	**0.49**	17
$mGlu_5$	6x54	V788A (r)	VU0415051	(P)	**0.19, 0.05**	15
$mGlu_5$	6x54	V788A (r)	VU29	(P)	**2.2**	17
$mGlu_5$	6x57	Y791A (r)	[^{3}H]Fenobam	(N)	**4.0**	19
$mGlu_5$	6x57	Y791A (r)	[^{3}H]MPEP	(N)	**4.9**	20
$mGlu_5$	6x57	Y792A (h)	2-BisPEB	(N)	*2.0*	18
$mGlu_5$	6x57	Y792F (h)	2-BisPEB	(N)	*1.4*	18
$mGlu_5$	6x57	Y792A (h)	3-BisPEB	(N)	*>20*	18
$mGlu_5$	6x57	Y792F (h)	3-BisPEB	(N)	*1.1*	18
$mGlu_5$	6x57	Y792A (h)	4-BisPEB	(N)	*3.0*	18
$mGlu_5$	6x57	Y792F (h)	4-BisPEB	(N)	*0.76*	18
$mGlu_5$	6x57	Y791F (r)	CPPHA	(P)	*No significant effect*	1
$mGlu_5$	6x57	Y791A (r)	DFB	(P)	*Potency increase abolished - efficacy increase remained*	21
$mGlu_5$	6x57	Y791A (r)	DPFE	(P)	**0.31**	17
$mGlu_5$	6x57	Y791F (r)	DPFE	(P)	*No significant effect*	17
$mGlu_5$	6x57	Y791A (r)	Fenobam	(N)	*>71*	19
$mGlu_5$	6x57	Y791A (r)	M-5MPEP	(N)	**1.5**	17
$mGlu_5$	6x57	Y791F (r)	M-5MPEP	(N)	*No significant effect*	17
$mGlu_5$	6x57	Y791A (r)	MPEP	(N)	*22.4*	20
$mGlu_5$	6x57	Y791A (r)	MPEP	(N)	**7.6**	17
$mGlu_5$	6x57	Y791F (r)	MPEP	(N)	*No significant effect*	15
$mGlu_5$	6x57	Y792A (h)	MPEP	(N)	*8.1*	18
$mGlu_5$	6x57	Y792F (h)	MPEP	(N)	*1.6*	18
$mGlu_5$	6x57	Y791A (r)	VU0285683	(N)	**10.7**	17
$mGlu_5$	6x57	Y791F (r)	VU0285683	(N)	*No significant effect*	17
$mGlu_5$	6x57	Y791A (r)	VU0366058	(N)	**0.38**	17
$mGlu_5$	6x57	Y791F (r)	VU0366058	(N)	*No significant effect*	17
$mGlu_5$	6x57	Y791A (r)	VU0366248	(N)	**12.3**	17
$mGlu_5$	6x57	Y791F (r)	VU0366248	(N)	*No significant effect*	17
$mGlu_5$	6x57	Y791A (r)	VU0366249	(N)	**20.4**	17
$mGlu_5$	6x57	Y791F (r)	VU0366249	(N)	*No significant effect*	17

mGlu$_5$	6x57	Y791A (r)	VU0409106 (N)	**12.6**	17
mGlu$_5$	6x57	Y791F (r)	VU0409106 (N)	*No significant effect*	17
mGlu$_5$	6x57	Y791A (r)	VU29 (P)	**2.0**	17
mGlu$_5$	6x57	Y791F (r)	VU29 (P)	*No significant effect*	17
mGlu$_5$	6x58	F792A (r)	[^{3}H]methoxyPEPy (N)	**1.3**	15
mGlu$_5$	6x58	F792Y (r)	CPPHA (P)	*No significant effect*	1
mGlu$_5$	6x58	F792A (r)	DPFE (P)	**0.28**	17
mGlu$_5$	6x58	F792A (r)	M-5MPEP (N)	*No significant effect*	17
mGlu$_5$	6x58	F792A (r)	MPEP (N)	**0.45, 0.93**	15
mGlu$_5$	6x58	F792A (r)	VU0285683 (N)	*No significant effect*	17
mGlu$_5$	6x58	F792A (r)	VU0360172 (P)	**0.27, 0.32**	15
mGlu$_5$	6x58	F792A (r)	VU0360173 (P)	**4.5, 0.28**	15
mGlu$_5$	6x58	F792A (r)	VU0366058 (N)	*No significant effect*	17
mGlu$_5$	6x58	F792A (r)	VU0366248 (N)	**1.4**	17
mGlu$_5$	6x58	F792A (r)	VU0366249 (N)	*No significant effect*	17
mGlu$_5$	6x58	F792A (r)	VU0403602 (P)	**1.3, 2.6**	15
mGlu$_5$	6x58	F792A (r)	VU0405386 (P)	**10.5, 1.9**	15
mGlu$_5$	6x58	F792A (r)	VU0405398 (P)	**0.40, 0.20**	15
mGlu$_5$	6x58	F792A (r)	VU0409106 (N)	**3.1**	17
mGlu$_5$	6x58	F792A (r)	VU0415051 (P)	**0.19, 0.62**	15
mGlu$_5$	6x58	F792A (r)	VU29 (P)	**0.3**	17
mGlu$_5$	6x59	G793V (r)	CPPHA (P)	*No significant effect*	1
mGlu$_5$	ECL3	S794T (r)	CPPHA (P)	*No significant effect*	1
mGlu$_5$	7x33	M802T (h)	[^{3}H]M-MPEP (N)	**1.1**	8
mGlu$_5$	7x33	M801T (r)	CDPPB (P)	*No significant effect*	23
mGlu$_5$	7x33	M802T (h)	CPCCOEt (N)	*Gain of inhibition -*<br>*(does not inhibit WT human mGlu$_5$)*	24
mGlu$_5$	7x33	M801T (r)	DFB (P)	*Abolished potentiation*	21
mGlu$_5$	7x33	M801T (r)	Fenobam (N)	*1.6*	19
mGlu$_5$	7x33	M801T (r)	MPEP (N)	*2.0*	20
mGlu$_5$	7x33	M801T (r)	VU29 (P)	*No significant effect*	23
mGlu$_5$	7x35	F803V (r)	CPPHA (P)	*No significant effect*	1
mGlu$_5$	7x35	F803V (r)	M-5MPEP (N)	*No significant effect*	17
mGlu$_5$	7x35	F803V (r)	VU0285683 (N)	*No significant effect*	17
mGlu$_5$	7x35	F803V (r)	VU0366058 (N)	*No significant effect*	17
mGlu$_5$	7x35	F803V (r)	VU0366248 (N)	*No significant effect*	17
mGlu$_5$	7x35	F803V (r)	VU0366249 (N)	*No significant effect*	17
mGlu$_5$	7x35	F803V (r)	VU0409106 (N)	*No significant effect*	17
mGlu$_5$	7x35	F803V (r)	VU29 (P)	*No significant effect*	17
mGlu$_5$	7x36	S805A (h)	[^{3}H]M-MPEP (N)	**1.9**	8
mGlu$_5$	7x36	S805A (h)	CPCCOEt (N)	*No gain of inhibition -*<br>*(does not inhibit WT human mGlu$_5$)*	24
mGlu$_5$	7x38	S806A (r)	[^{3}H]methoxyPEPy (N)	**3.4**	15

mGlu$_5$	7x38	S806A (r)	DPFE (P)	*No significant effect*	17
mGlu$_5$	7x38	S806A (r)	M-5MPEP (N)	*No significant effect*	17
mGlu$_5$	7x38	S806A (r)	MPEP (N)	**1.7, 4.6**	15
mGlu$_5$	7x38	S806T (r)	MPEP (N)	*No significant effect*	15
mGlu$_5$	7x38	S806A (r)	VU0285683 (N)	*No significant effect*	17
mGlu$_5$	7x38	S806A (r)	VU0366058 (N)	*No significant effect*	17
mGlu$_5$	7x38	S806A (r)	VU0366248 (N)	*No significant effect*	17
mGlu$_5$	7x38	S806A (r)	VU0366249 (N)	*No significant effect*	17
mGlu$_5$	7x38	S806A (r)	VU0405386 (P)	**5.6**	15
mGlu$_5$	7x38	S806A (r)	VU0409106 (N)	*No significant effect*	17
mGlu$_5$	7x38	S806A (r)	VU29 (P)	*No significant effect*	17
mGlu$_5$	7x40	S808A (r)	[^{3}H]methoxyPEPy (N)	**Abolished binding**	15
mGlu$_5$	7x40	S808T (r)	[^{3}H]methoxyPEPy (N)	**Abolished binding**	15
mGlu$_5$	7x40	S809A (h)	2-BisPEB (N)	*19*	18
mGlu$_5$	7x40	S809F (h)	2-BisPEB (N)	*63*	18
mGlu$_5$	7x40	S809A (h)	3-BisPEB (N)	*1.1*	18
mGlu$_5$	7x40	S809F (h)	3-BisPEB (N)	*2.9*	18
mGlu$_5$	7x40	S809A (h)	4-BisPEB (N)	*0.48*	18
mGlu$_5$	7x40	S809F (h)	4-BisPEB (N)	*2.4*	18
mGlu$_5$	7x40	S808A (r)	DPFE (P)	**2.9**	17
mGlu$_5$	7x40	S808T (r)	DPFE (P)	**2.0**	17
mGlu$_5$	7x40	S808A (r)	M-5MPEP (N)	**263.0**	17
mGlu$_5$	7x40	S808T (r)	M-5MPEP (N)	**57.5**	17
mGlu$_5$	7x40	S808A (r)	MPEP (N)	**39.8**	15
mGlu$_5$	7x40	S808T (r)	MPEP (N)	**47.9**	15
mGlu$_5$	7x40	S809A (h)	MPEP (N)	*52*	18
mGlu$_5$	7x40	S809F (h)	MPEP (N)	*490*	18
mGlu$_5$	7x40	S808A (r)	VU0285683 (N)	**10.2**	17
mGlu$_5$	7x40	S808T (r)	VU0285683 (N)	**21.4**	17
mGlu$_5$	7x40	S808A (r)	VU0360172 (P)	**3.4**	15
mGlu$_5$	7x40	S808T (r)	VU0360172 (P)	**1.2**	15
mGlu$_5$	7x40	S808A (r)	VU0360173 (P)	**Abolished potentiation**	15
mGlu$_5$	7x40	S808T (r)	VU0360173 (P)	**2.6**	15
mGlu$_5$	7x40	S808A (r)	VU0366058 (N)	**5.3**	17
mGlu$_5$	7x40	S808T (r)	VU0366058 (N)	**1.5**	17
mGlu$_5$	7x40	S808A (r)	VU0366248 (N)	**10.7**	17
mGlu$_5$	7x40	S808T (r)	VU0366248 (N)	**0.14**	17
mGlu$_5$	7x40	S808A (r)	VU0366249 (N)	**Abolished inhibition**	17
mGlu$_5$	7x40	S808T (r)	VU0366249 (N)	**0.37**	17
mGlu$_5$	7x40	S808A (r)	VU0403602 (P)	**45.7**	15
mGlu$_5$	7x40	S808T (r)	VU0403602 (P)	**9.5**	15
mGlu$_5$	7x40	S808A (r)	VU0405386 (P)	**3.6**, *PAM to neutral switch*	15
mGlu$_5$	7x40	S808T (r)	VU0405386 (P)	**0.98**	15

mGlu$_5$	7x40	S808A (r)	VU0405398 (P)	**4.6**, *PAM to NAM switch*	[15]
mGlu$_5$	7x40	S808T (r)	VU0405398 (P)	**3.5**	[15]
mGlu$_5$	7x40	S808A (r)	VU0409106 (N)	**0.76**	[17]
mGlu$_5$	7x40	S808T (r)	VU0409106 (N)	**7.8**	[17]
mGlu$_5$	7x40	S808A (r)	VU0415051 (P)	**1.4**	[15]
mGlu$_5$	7x40	S808T (r)	VU0415051 (P)	**9.5**	[15]
mGlu$_5$	7x40	S808A (r)	VU0424465 (P)	**10.0**	[22]
mGlu$_5$	7x40	S808A (r)	VU0430644 (P)	*PAM to NAM switch*	[22]
mGlu$_5$	7x40	S808A (r)	VU0430644 (P)	**6.5**	[22]
mGlu$_5$	7x40	S808A (r)	VU0465731 (P)	**42.7**	[22]
mGlu$_5$	7x40	S808A (r)	VU29 (P)	**0.7**	[17]
mGlu$_5$	7x40	S808T (r)	VU29 (P)	**1.5**	[17]
mGlu$_5$	7x41	A809V (r)	[^{3}H]Fenobam (N)	**Abolished binding**	[19]
mGlu$_5$	7x41	A810V (h)	[^{3}H]M-MPEP (N)	**Abolished binding**	[8]
mGlu$_5$	7x41	A809G (r)	[^{3}H]methoxyPEPy (N)	**Abolished binding**	[15]
mGlu$_5$	7x41	A809V (r)	[^{3}H]methoxyPEPy (N)	**Abolished binding**	[15]
mGlu$_5$	7x41	A809V (r)	[^{3}H]MPEP (N)	**Abolished binding**	[20]
mGlu$_5$	7x41	A810V (h)	2-BisPEB (N)	*10*	[18]
mGlu$_5$	7x41	A810V (h)	3-BisPEB (N)	*>20*	[18]
mGlu$_5$	7x41	A810V (h)	4-BisPEB (N)	*3.6*	[18]
mGlu$_5$	7x41	A809V (r)	CDPPB (P)	*Abolished potentiation*	[23]
mGlu$_5$	7x41	A809V (r)	CPPHA (P)	*No significant effect*	[1]
mGlu$_5$	7x41	A809V (r)	DFB (P)	*Potency increase abolished - efficacy increase remained*	[21]
mGlu$_5$	7x41	A809G (r)	DPFE (P)	**10.7**	[17]
mGlu$_5$	7x41	A809V (r)	DPFE (P)	**6.5**	[17]
mGlu$_5$	7x41	A809V (r)	Fenobam (N)	*23.0*	[19]
mGlu$_5$	7x41	A809G (r)	M-5MPEP (N)	**43.7**	[17]
mGlu$_5$	7x41	A809V (r)	M-5MPEP (N)	**2.1**	[17]
mGlu$_5$	7x41	A809G (r)	MPEP (N)	**25.1**	[15]
mGlu$_5$	7x41	A809V (r)	MPEP (N)	*Decreased inhibition*	[15]
mGlu$_5$	7x41	A809V (r)	MPEP (N)	**114.8**	[16]
mGlu$_5$	7x41	A809V (r)	MPEP (N)	*45.0*	[20]
mGlu$_5$	7x41	A810V (h)	MPEP (N)	*63*	[18]
mGlu$_5$	7x41	A809G (r)	VU0285683 (N)	**13.2**	[17]
mGlu$_5$	7x41	A809V (r)	VU0285683 (N)	**4.8**	[17]
mGlu$_5$	7x41	A809G (r)	VU0360172 (P)	**5.6**	[15]
mGlu$_5$	7x41	A809V (r)	VU0360172 (P)	**11.0**	[15]
mGlu$_5$	7x41	A809G (r)	VU0360173 (P)	**2.1**	[15]
mGlu$_5$	7x41	A809V (r)	VU0360173 (P)	**Abolished potentiation**	[15]
mGlu$_5$	7x41	A809G (r)	VU0366058 (N)	**12.9**	[17]
mGlu$_5$	7x41	A809V (r)	VU0366058 (N)	**2.40**	[17]
mGlu$_5$	7x41	A809G (r)	VU0366248 (N)	**9.3**	[17]

mGlu$_5$	7x41	A809V (r)	VU0366248 (N)	**27.5**	[17]
mGlu$_5$	7x41	A809G (r)	VU0366249 (N)	**Abolished inhibition**	[17]
mGlu$_5$	7x41	A809V (r)	VU0366249 (N)	**Abolished inhibition**	[17]
mGlu$_5$	7x41	A809G (r)	VU0403602 (P)	**34.7**	[15]
mGlu$_5$	7x41	A809V (r)	VU0403602 (P)	**40.7**	[15]
mGlu$_5$	7x41	A809G (r)	VU0405386 (P)	**27.5**	[15]
mGlu$_5$	7x41	A809V (r)	VU0405386 (P)	**66.1**	[15]
mGlu$_5$	7x41	A809G (r)	VU0405398 (P)	**11.5**	[15]
mGlu$_5$	7x41	A809V (r)	VU0405398 (P)	**Abolished potentiation**	[15]
mGlu$_5$	7x41	A809G (r)	VU0409106 (N)	**1.8**	[17]
mGlu$_5$	7x41	A809V (r)	VU0409106 (N)	**23.4**	[17]
mGlu$_5$	7x41	A809G (r)	VU0415051 (P)	**11.5**	[15]
mGlu$_5$	7x41	A809V (r)	VU0415051 (P)	**60.3**	[15]
mGlu$_5$	7x41	A809V (r)	VU29 (P)	**36.3**	[16]
mGlu$_5$	7x41	A809V (r)	VU29 (P)	*Abolished potentiation*	[23]
mGlu$_5$	7x41	A809G (r)	VU29 (P)	**11.8**	[17]
mGlu$_5$	7x41	A809V (r)	VU29 (P)	**36.3**	[17]
mGlu$_5$	7x42	T810A (r)	[^{3}H]methoxyPEPy (N)	**2.4**	[15]
mGlu$_5$	7x42	T810A (r)	DPFE (P)	*No significant effect*	[17]
mGlu$_5$	7x42	T810A (r)	M-5MPEP (N)	*No significant effect*	[17]
mGlu$_5$	7x42	T810A (r)	MPEP (N)	**3.9**	[15]
mGlu$_5$	7x42	T810S (r)	MPEP (N)	*No significant effect*	[15]
mGlu$_5$	7x42	T810A (r)	VU0285683 (N)	*No significant effect*	[17]
mGlu$_5$	7x42	T810A (r)	VU0366058 (N)	*No significant effect*	[17]
mGlu$_5$	7x42	T810A (r)	VU0366248 (N)	*No significant effect*	[17]
mGlu$_5$	7x42	T810A (r)	VU0366249 (N)	*No significant effect*	[17]
mGlu$_5$	7x42	T810A (r)	VU0409106 (N)	*No significant effect*	[17]
mGlu$_5$	7x42	T810A (r)	VU29 (P)	*No significant effect*	[17]
mGlu$_5$	7x44	A813F (h)	2-BisPEB (N)	*1.5*	[18]
mGlu$_5$	7x44	A813F (h)	3-BisPEB (N)	*1.5*	[18]
mGlu$_5$	7x44	A813F (h)	4-BisPEB (N)	*1.7*	[18]
mGlu$_5$	7x44	A812S (r)	MPEP (N)	*No significant effect*	[15]
mGlu$_5$	7x44	A813F (h)	MPEP (N)	*1.2*	[18]
mGlu$_5$	7x45	L813A (r)	MPEP (N)	*No significant effect*	[15]
mGlu$_5$	7x47	C815A (r)	[^{3}H]methoxyPEPy (N)	**1.7**	[15]
mGlu$_5$	7x47	C815A (r)	DPFE (P)	*No significant effect*	[17]
mGlu$_5$	7x47	C815A (r)	M-5MPEP (N)	*No significant effect*	[17]
mGlu$_5$	7x47	C815A (r)	MPEP (N)	**2.0, 1.7**	[15]
mGlu$_5$	7x47	C815A (r)	VU0285683 (N)	*No significant effect*	[17]
mGlu$_5$	7x47	C815A (r)	VU0360172 (P)	**1.3, 0.68**	[15]
mGlu$_5$	7x47	C815A (r)	VU0360173 (P)	**4.4, 1.3**	[15]
mGlu$_5$	7x47	C815A (r)	VU0366058 (N)	*No significant effect*	[17]
mGlu$_5$	7x47	C815A (r)	VU0366248 (N)	*No significant effect*	[17]

$mGlu_5$	7x47	C815A (r)	VU0366249	(N)	*No significant effect*	[17]
$mGlu_5$	7x47	C815A (r)	VU0403602	(P)	**6.9, 1.8**	[15]
$mGlu_5$	7x47	C815A (r)	VU0405386	(P)	**2.6, 1.0**	[15]
$mGlu_5$	7x47	C815A (r)	VU0405398	(P)	**1.6, 0.62**	[15]
$mGlu_5$	7x47	C815A (r)	VU0409106	(N)	*No significant effect*	[17]
$mGlu_5$	7x47	C815A (r)	VU0415051	(P)	**1.2, 0.91**	[15]
$mGlu_5$	7x47	C815A (r)	VU29	(P)	*No significant effect*	[17]
$mGlu_5$	7x48	M816A (r)	DPFE	(P)	*No significant effect*	[17]
$mGlu_5$	7x48	M816A (r)	M-5MPEP	(N)	*No significant effect*	[17]
$mGlu_5$	7x48	M816A (r)	MPEP	(N)	*No significant effect*	[15]
$mGlu_5$	7x48	M816A (r)	VU0285683	(N)	*No significant effect*	[17]
$mGlu_5$	7x48	M816A (r)	VU0366058	(N)	*No significant effect*	[17]
$mGlu_5$	7x48	M816A (r)	VU0366248	(N)	*No significant effect*	[17]
$mGlu_5$	7x48	M816A (r)	VU0366249	(N)	*No significant effect*	[17]
$mGlu_5$	7x48	M816A (r)	VU0409106	(N)	*No significant effect*	[17]
$mGlu_5$	7x48	M816A (r)	VU29	(P)	*No significant effect*	[17]
$mGlu_5$	7x49	V819T (h)	[^{3}H]M-MPEP	(N)	**1.2**	[8]
$mGlu_5$	7x52	V822M (h)	[^{3}H]M-MPEP	(N)	**0.89**	[8]
$mGlu_5$	7x56	L826I (h)	[^{3}H]M-MPEP	(N)	**0.89**	[8]

Supplementary Table 2: Docking pose selection for RO5488608

The first 10 poses of RO5488608 from induced fit docking. [1]RMSD values on all ligand heavy atoms relative to the higher-ranking pose given in parentheses. [2]Score values from the induced fit docking protocol (2014-2, Glide version 6.1, Prime version 3.4, Schrödinger, LLC, New York, NY, 2014)[25]. [3]Number of hydrogen bonds and bad van der Waals (vdW) interactions between the ligand pose and the receptor as defined in Maestro (version 9.8, Schrödinger, LLC, New York, NY, 2014); "Glide" settings with a distance cut-off of 2.8 Å for hydrogen bonds and default for vdW contacts. [4]Difference in conformational energy between the docking pose and the global energy minimum conformation. [5]Interaction observed in the docking output pose to the residues known from mutational data to be important for RO5488608 inhibition. Criteria that exclude or favour pose selection are coloured red and green, respectively.

**Pose**	**[1]RMSD (to pose)**	**[2]IFD score**	**[2]gscore**	**[2]Emodel**	**[3]H-bond**	**[3]Clashes**	**[4]ΔE**	**[5]R3x33**	**[5]H45x52**	**[5]L5x44**	**[5]V7x37**
1		-463.7	-13.5	-122.9	2	5	4.4	vdW	h-bond + aromatic	vdW	vdW
2		-463.6	-13.8	-99.5	6	7	4.5	vdW	h-bond + aromatic	vdW	No contacts
3		-463.6	-14.3	-82.7	4	14	3.7	No contacts	h-bond	vdW	vdW
4	0.24 (2)										
5	0.77 (2)										
6	0.61 (1)										
7		-463.1	-13.2	-120.9	10	8	0.7	vdW	h-bond + aromatic	vdW	vdW
8	0.20 (7)										
9		-462.9	-13.8	-125.4	7	17	3.6	vdW	h-bond + aromatic	vdW	vdW
10	0.11 (9)										

Induced fit docking of RO5488608 to the $mGlu_2$ homology model resulted in 19 output poses and among the top 10 ranked poses that were further evaluated five had RMSD values relative to higher ranked poses below 0.8 Å and were considered duplicates. The five unique poses had similar IFD scores and gscores while pose 2 and 3 display lower Emodel scores. Furthermore, pose 2 and 3 each lack interactions to one of the residues shown by mutational studies to be important for RO5488608 inhibition[10], V7x37 and R3x33, respectively, and are disregarded as likely binding modes. Of the three remaining poses, pose 7, the 4th ranked unique pose, is selected as the most likely binding pose mainly due to better hydrogen bonding interactions but additionally it has the lowest ΔE.

Supplementary Table 3: Docking pose selection of ML337

The first 10 poses of ML337 from induced fit docking. [1]RMSD values on all ligand heavy atoms relative to the higher-ranking pose given in parentheses. [2]Score values from the induced fit docking protocol (2014-2, Glide version 6.1, Prime version 3.4, Schrödinger, LLC, New York, NY, 2014)[25]. [3]Number of hydrogen bonds and bad vdW interactions between the ligand pose and the receptor as defined in Maestro (version 9.8, Schrödinger, LLC, New York, NY, 2014); "Glide" settings with a distance cut-off of 2.8 Å for hydrogen bonds and default for vdW contacts. [4]Difference in conformational energy between the docking pose and the global energy minimum conformation. [5]Fit to the SAR information from ML337 analogues. Criteria that exclude or favour pose selection are coloured red and green, respectively.

Pose	[1]RMSD (to pose)	[2]IFD score	[2]gscore	[2]Emodel	^{3}H-bond	[3]Clashes	[4]ΔE	[5]SAR
1		-450.3	-11.0	-73.5	1	10	6.8	Not in position to accept h-bond
2		-450.2	-10.9	-75.3	0	11	0.2	MeOPh not in narrow pocket
3	0.48 (2)							
4	0.76 (2)							
5	0.36 (2)							
6		-450.0	-11.1	-81.6	2	10	5.3	OK
7	0.48 (1)							
8		-449.5	-10.8	-69.1	1	11	0.0	Not in position to accept h-bond
9		-449.4	-10.7	-70.4	1	8	4.9	Not in position to accept h-bond
10	0.67 (1)							

Induced fit docking of ML337 to the $mGlu_3$ homology model resulted in 18 output poses and among the top 10 ranked poses that were further evaluated five had RMSD values relative to higher ranked poses below 0.8 Å and were considered duplicates. The five unique poses had similar IFD scores and gscores while the Emodel score is markedly better for pose 6 relative to the other four. Pose 6 also has one or two additional hydrogen bonds compared to the other poses. The sparse SAR information from ML337 analogues[26, 27] shows that even small changes to the *p*-methoxyphenyl moiety markedly reduce the activity indicating a tight fit in the binding pocket. Additionally, the polar group of the piperidine moiety (hydroxy of ML337) is also important for the activity and a methoxy analogue show that a hydrogen bonding acceptor function is sufficient to retain inhibition. Based on this poses 1, 2, 8 and 9 are disregarded and we select pose 6, the 3rd ranked unique pose, as the most likely binding pose.

Supplementary Table 4: Docking pose selection of MMPIP

The poses of MMPIP from induced fit docking. [1]RMSD values on all ligand heavy atoms relative to the higher-ranking pose given in parentheses. [2]Score values from the induced fit docking protocol (2014-2, Glide version 6.1, Prime version 3.4, Schrödinger, LLC, New York, NY, 2014)[25]. [3]Number of hydrogen bonds and bad vdW interactions between the ligand pose and the receptor as defined in Maestro (version 9.8, Schrödinger, LLC, New York, NY, 2014); "Glide" settings with a distance cut-off of 2.8 Å for hydrogen bonds and default for vdW contacts. [4]Difference in conformational energy between the docking pose and the global energy minimum conformation. [5]Fit to the SAR information from MMPIP analogues. Criteria that exclude or favour pose selection are coloured red and green, respectively.

Pose	[1]RMSD (to pose)	[2]IFD score	[2]gscore	[2]Emodel	^{3}H-bond	[3]Clashes	[4]ΔE	[5]SAR	Q5x39	I5x40	V6x49	S5x47
1		-437.4	-9.9	-69.1	2	14	0.0	OK	vdW	vdW	vdW	h-bond
2		-437.2	-10.8	-67.5	1	14	0.1	OK	vdW	vdW	vdW	vdW
3	0.79 (1)											
4	0.52 (2)											

Induced fit docking of MMPIP to the $mGlu_7$ homology model resulted in only four output poses and two of these had RMSD values relative to higher ranked poses below 0.8 Å and were considered duplicates. The scoring values, ΔE, and steric clashes of the two remaining poses are too similar to allow a clear distinction between these two different poses. The number of hydrogen bonds favour pose 1 but to further support the selection we reviewed the limited SAR information available[28]. From this it can be derived that there should be a tight fit of the receptor pocket to the *N*-methyl substituent as small increases reduce activity markedly and the pyridine nitrogen should not have a specific hydrogen bond interaction to the receptor as a replacement with phenyl does not affect activity. Additionally, small substitutions are allowed in both the 3- and 4-positions on the phenyl of the 4-methoxyphenyl moiety. All three observations are compatible with both poses 1 and 2. As a last attempt to gain support for selecting pose 1 we looked at interactions to residues specific for group III/$mGlu_7$, Gln5x39, Ile5x40, Val6x49 and Ser5x47, as MMPIP is known to be selective for mGu_7 over all other seven mGlu subtypes. Both poses show vdW contacts to the three first-mentioned residues but only pose 1 display a specific hydrogen bond to the group III unique Ser5x47, which is likely part of the explanation for the observed selectivity profile. Though there I no clear distinction between pose 1 and 2 in the selection criteria used we select pose 1 as the most likely binding mode of MMPIP based on the additional hydrogen bond to the receptor supported by the likely contribution of this interaction to the $mGlu_7$ selectivity.

Supplementary Table 5: Similarity matrix of the mGlu receptors based on the 7TM domain and the binding site residues

	$mGlu_1$	$mGlu_5$	$mGlu_2$	$mGlu_3$	$mGlu_4$	$mGlu_6$	$mGlu_7$	$mGlu_8$
$mGlu_1$		87	68	68	65	68	65	65
$mGlu_5$	87		68	68	74	74	74	74
$mGlu_2$	72	70		97	77	74	77	81
$mGlu_3$	68	68	88		71	71	71	74
$mGlu_4$	69	71	75	70		90	94	97
$mGlu_6$	64	67	73	68	85		87	90
$mGlu_7$	67	69	75	70	92	87		97
$mGlu_8$	67	70	75	72	93	87	94	

Similarity matrix for the mGlu receptors. Percentage similarity of the 7TM domain (lower left part of the table) and the binding site residues (upper right part of the table) for the eight mGlu receptors as calculated with GPCRDB Tools[29].

Supplementary Table 6: Similarity between the mGlu crystal structures and other selected class C GPCRs

	$mGlu_1$	$mGlu_5$	CaS	TAS1R1	$GPRC_6$	$GABA_{B1}$
$mGlu_1$			52	39	35	39
$mGlu_5$			52	35	32	42
CaS	50	52				
TAS1R1	45	41				
$GPRC_6$	45	44				
$GABA_{B1}$	39	39				

Similarity matrix for class C GPCRs. Percentage similarity of the 7TM domain (lower left part of the table) and the binding site residues (upper right part of the table) for selected class C GPCRs as calculated with GPCRDB Tools[29].

References

1. Chen, Y., Goudet, C., Pin, J. P. & Conn, P. J. *N*-{4-Chloro-2-[(1,3-dioxo-1,3-dihydro-2*H*-isoindol-2-yl)methyl]phenyl}-2-hydroxybe nzamide (CPPHA) acts through a novel site as a positive allosteric modulator of group 1 metabotropic glutamate receptors. *Mol. Pharmacol.* **73**, 909-918 (2008).

2. Wu, H. *et al.* Structure of a class C GPCR metabotropic glutamate receptor 1 bound to an allosteric modulator. *Science* **344**, 58-64 (2014).

3. Malherbe, P. *et al.* Mutational analysis and molecular modeling of the allosteric binding site of a novel, selective, noncompetitive antagonist of the metabotropic glutamate 1 receptor. *J. Biol. Chem.* **278**, 8340-8347 (2003).

4. Fukuda, J. *et al.* Identification of a novel transmembrane domain involved in the negative modulation of mGluR1 using a newly discovered allosteric mGluR1 antagonist, 3-cyclohexyl-5-fluoro-6-methyl-7-(2-morpholin-4-ylethoxy)-4*H*-chromen-4-one. *Neuropharmacology* **57**, 438-445 (2009).

5. Knoflach, F. *et al.* Positive allosteric modulators of metabotropic glutamate 1 receptor: characterization, mechanism of action, and binding site. *Proc. Natl. Acad. Sci. U.S.A.* **98**, 13402-13407 (2001).

6. Hemstapat, K. *et al.* A novel class of positive allosteric modulators of metabotropic glutamate receptor subtype 1 interact with a site distinct from that of negative allosteric modulators. *Mol. Pharmacol.* **70**, 616-626 (2006).

7. Suzuki, G. *et al.* Pharmacological characterization of a new, orally active and potent allosteric metabotropic glutamate receptor 1 antagonist, 4-[1-(2-fluoropyridin-3-yl)-5-methyl-1*H*-1,2,3-triazol-4-yl]-*N*-isopropyl-*N*-methyl- 3,6-dihydropyridine-1(2*H*)-carboxamide (FTIDC). *J. Pharmacol. Exp. Ther.* **321**, 1144-1153 (2007).

8. Pagano, A. *et al.* The non-competitive antagonists 2-methyl-6-(phenylethynyl)pyridine and 7-hydroxyiminocyclopropan[*b*]chromen-1a-carboxylic acid ethyl ester interact with overlapping binding pockets in the transmembrane region of group I metabotropic glutamate receptors. *J. Biol. Chem.* **275**, 33750-33758 (2000).

9. Farinha, A. *et al.* Molecular determinants of positive allosteric modulation of the human metabotropic glutamate receptor 2. *Br. J. Pharmacol.* **172**, 2383-2396 (2015).

10. Lundstrom, L. *et al.* Structural determinants of allosteric antagonism at metabotropic glutamate receptor 2: mechanistic studies with new potent negative allosteric modulators. *Br. J. Pharmacol.* **164**, 521-537 (2011).

11. Schaffhauser, H. *et al.* Pharmacological characterization and identification of amino acids involved in the positive modulation of metabotropic glutamate receptor subtype 2. *Mol. Pharmacol.* **64**, 798-810 (2003).

12. Rowe, B. A. *et al.* Transposition of three amino acids transforms the human metabotropic glutamate receptor (mGluR)-3-positive allosteric modulation site to mGluR2, and additional characterization of the mGluR2-positive allosteric modulation site. *J. Pharmacol. Exp. Ther.* **326**, 240-251 (2008).

13. Hemstapat, K. *et al.* A novel family of potent negative allosteric modulators of group II metabotropic glutamate receptors. *J. Pharmacol. Exp. Ther.* **322**, 254-264 (2007).

14. Rovira, X. *et al.* Overlapping binding sites drive allosteric agonism and positive cooperativity in type 4 metabotropic glutamate receptors. *FASEB J.* **29**, 116-130 (2015).

15. Gregory, K. J. *et al.* Probing the metabotropic glutamate receptor 5 ($mGlu_5$) positive allosteric modulator (PAM) binding pocket: discovery of point mutations that engender a "molecular switch" in PAM pharmacology. *Mol. Pharmacol.* **83**, 991-1006 (2013).

16. Gregory, K. J. *et al.* Investigating metabotropic glutamate receptor 5 allosteric modulator cooperativity, affinity, and agonism: enriching structure-function studies and structure-activity relationships. *Mol. Pharmacol.* **82**, 860-875 (2012).

17. Gregory, K. J. *et al.* Identification of specific ligand-receptor interactions that govern binding and cooperativity of diverse modulators to a common metabotropic glutamate receptor 5 allosteric site. *ACS Chem. Neurosci.* **5**, 282-295 (2014).

18. Mølck, C. *et al.* Pharmacological characterization and modeling of the binding sites of novel 1,3-bis(pyridinylethynyl)benzenes as metabotropic glutamate receptor 5-selective negative allosteric modulators. *Mol. Pharmacol.* **82**, 929-937 (2012).

19. Malherbe, P. *et al.* Comparison of the binding pockets of two chemically unrelated allosteric antagonists of the mGlu5 receptor and identification of crucial residues involved in the inverse agonism of MPEP. *J. Neurochem.* **98**, 601-615 (2006).

20. Malherbe, P. *et al.* Mutational analysis and molecular modeling of the binding pocket of the metabotropic glutamate 5 receptor negative modulator 2-methyl-6-(phenylethynyl)-pyridine. *Mol. Pharmacol.* **64**, 823-832 (2003).

21. Muhlemann, A. *et al.* Determination of key amino acids implicated in the actions of allosteric modulation by 3,3'-difluorobenzaldazine on rat mGlu5 receptors. *Eur. J. Pharmacol.* **529**, 95-104 (2006).

22. Turlington, M. *et al.* Exploration of allosteric agonism structure-activity relationships within an acetylene series of metabotropic glutamate receptor 5 ($mGlu_5$) positive allosteric modulators (PAMs): discovery of 5-((3-fluorophenyl)ethynyl)-*N*-(3-methyloxetan-3-yl)picolinamide (ML254). *J. Med. Chem.* **56**, 7976-7996 (2013).

23. Chen, Y. *et al.* Interaction of novel positive allosteric modulators of metabotropic glutamate receptor 5 with the negative allosteric antagonist site is required for potentiation of receptor responses. *Mol. Pharmacol.* **71**, 1389-1398 (2007).

24. Litschig, S. *et al.* CPCCOEt, a noncompetitive metabotropic glutamate receptor 1 antagonist, inhibits receptor signaling without affecting glutamate binding. *Mol. Pharmacol.* **55**, 453-461 (1999).

25. Sherman, W., Day, T., Jacobson, M. P., Friesner, R. A. & Farid, R. Novel procedure for modeling ligand/receptor induced fit effects. *J. Med. Chem.* **49**, 534-553 (2006).

26. Sheffler, D. J. *et al.* Development of a novel, CNS-penetrant, metabotropic glutamate receptor 3 (mGlu3) NAM probe (ML289) derived from a closely related mGlu5 PAM. *Bioorg. Med. Chem. Lett.* **22**, 3921-3925 (2012).

27. Wenthur, C. J. *et al.* Discovery of (*R*)-(2-fluoro-4-((-4-methoxyphenyl)ethynyl)phenyl) (3-hydroxypiperidin-1-yl)methanone (ML337), an $mGlu_3$ selective and CNS penetrant negative allosteric modulator (NAM). *J. Med. Chem.* **56**, 5208-5212 (2013).

28. Nakamura, M. *et al.* Isoxazolopyridone derivatives as allosteric metabotropic glutamate receptor 7 antagonists. *Bioorg. Med. Chem. Lett.* **20**, 726-729 (2010).

29. Isberg, V. *et al.* GPCRDB: an information system for G protein-coupled receptors. *Nucleic. Acids Res.* **42**, D422-425 (2014).
